# Hardy Spaces and Canonical Kernels on Quadric CR Manifolds

**DOI:** 10.1007/s12220-024-01708-4

**Published:** 2024-06-10

**Authors:** Albert Boggess, Jennifer Brooks, Andrew Raich

**Affiliations:** 1 School of Mathematical and Statistical Sciences, Arizona State University, Physical Sciences Building A-Wing Rm. 216 901 S. Palm Walk, Tempe, AZ 85287-1804 USA; 2 Brigham Young University - 352 TMCB, Provo, UT 84602 USA; 3 Department of Mathematical Sciences, 1 University of Arkansas, SCEN 326, Fayetteville, AR 72701 USA

**Keywords:** Quadric submanifolds, Higher codimension, Hardy spaces, Bergman projection, Bergman kernel, Szegö projection, Szegö kernel, 32A25, 32A35, 32A36, 32A40, 32V25

## Abstract

CR functions on an embedded quadric *M* always extend holomorphically to $M+i\Gamma _M$ where $\Gamma _M$ is the closure of the convex hull of the image of the Levi form. When $\Gamma _M$ is a closed polygonal cone, we show that the Bergman kernel on the interior of $M+i\Gamma _M$ is a derivative of the Szegö kernel. Moreover, we develop the $L^p$ Hardy space theory which turns out to be particularly robust. We provide examples that show that it is unclear how to formulate a corresponding relationship between the Bergman and Szegö kernels on a wider class of quadrics.

## Introduction

A quadric manifold *M* is a CR manifold of the form

1 $$\begin{aligned} M = \{(z,w) \in \mathbb {C}^n\times \mathbb {C}^m : {{\,\textrm{Im}\,}}w = \phi (z,z)\} \end{aligned}$$

where $\phi :\mathbb {C}^n\times \mathbb {C}^n\rightarrow \mathbb {C}^m$ is a sesquilinear form. Associated to *M* are

$$\begin{aligned} \Gamma _{M} = \text {the closure of the convex hull of}~~\{\phi (z,z): z \in \mathbb {C}^n \} \end{aligned}$$

and the domain

$$\begin{aligned} \Omega _M=\text {int}(M+i\Gamma _M). \end{aligned}$$

It is well known that a CR function on *M* extends to $M+i\Gamma _M$ [2]. In this paper, we investigate a class of quadrics for which $\Gamma _M$ is a closed, polygonal cone, and our goals are twofold: First, we prove that the Bergman kernel on ${\text {int}}(M+i\Gamma _M)$ is an *m*th order derivative of the Szegö kernel. Second, we show that the $L^p$ Hardy space theory is unusually robust because the analytic discs with their boundaries in *M* have a particularly simple form.

The motivation for the first problem originates in an off-hand remark in the now-classic paper [8] on the Bergman and Szegö kernels for pseudoconvex domains of finite type in $\mathbb {C}^2$ (that is essentially repeated in [6]). Specifically, if

$$\begin{aligned} \Omega = \{ z=(z_1,z_2)\in \mathbb {C}^2: \textrm{Im}z_2 > P(z_1) \} \end{aligned}$$

for $P :\mathbb {C}\rightarrow \mathbb {R}$ a subharmonic, non-harmonic polynomial, then the Bergman kernel *B* and the Szegö kernel *S* satisfy

2 $$\begin{aligned} B(z,w)=2i \frac{\partial S}{\partial \bar{w}_2}(z,w). \end{aligned}$$

Nagel et al.’s proof of (2) uses the fact that the Bergman kernel is the unique function on $\Omega \times \Omega$ that is holomorphic in *z*, conjugate symmetric, and reproduces $A^2(\Omega )$, the class of holomorphic functions in $L^2(\Omega )$. The argument that integrating against $2i \frac{\partial S}{\partial \bar{w}_2}(z,w)$ reproduces holomorphic functions uses a clever integration by parts, though doing it rigorously requires knowledge of the behavior of functions in $A^2(\Omega )$.

Equation (2) is beguiling in its simplicity, and a goal of this paper is to determine when there is a higher codimension analogue of it. The additional complication in increasing from $m=1$ to $m\ge 2$ is that the structure of $\Gamma _M$ is vastly more complicated. For pseudoconvex domains in $\mathbb {C}^2$, $\Gamma _M = [0,\infty )$, while the structure can be much more complicated when $m\ge 2$, even for CR manifolds as “simple" as quadrics, as seen by the examples at the end of Sect. 2.

Our second goal is to develop the $L^p$ Hardy space theory for $H^p(\overline{\Omega })$. Although Boggess [3] established the $L^p$ Hardy space theory for a general quadric $M \subset \mathbb {C}^n\times \mathbb {C}^m$, it turns out that when $\Gamma _M$ is a polygonal cone, the Hardy space theory is particularly strong. This stems from the simple form of the analytic discs whose boundaries lie in *M*. For a general quadric, such a disc is a power series in the unit disc variable $\zeta$ whereas for the quadrics we study, we can fill out $M+i\Gamma _M$ with analytic discs that are polynomial in $\zeta$. This allows us to both simplify the arguments of [3] and improve the convergence for $1 \le p < \infty$.

One of the first results we prove is the existence of coordinates for $\mathbb {C}^n \times \mathbb {C}^m$ in which

$$\begin{aligned} \phi (z,z) = \sum _{j=1}^N |z_j|^2 V_j \end{aligned}$$

for a spanning set of vectors $V_1, \dots , V_N \in \Gamma _M \subset \mathbb {R}^m$ (Proposition 2.4). Nagel, Ricci, and Stein developed the notion of a flag kernel and flag singularity structure to study the harmonic analysis of these quadrics [7]. They established the $L^p$ mapping properties and pointwise estimates for the integral kernels of the objects associated to $\bar{\partial }_b$, including for the complex Green operator and the Szegö projection.

For additional background on the analysis of quadric submanifolds of $\mathbb {C}^n\times \mathbb {C}^m$, please see [5].

### Outline of Results

Throughout the manuscript, we assume that $$n\ge 1$$. We will assume that $$\Gamma _M$$ is closed, proper subset $$\mathbb {R}^m$$, and equal to the convex hull of a spanning set of vectors, $$V_1, \dots , V_N \in \mathbb {R}^m$$, where $$m \le N \le n$$. Note that *m* is the real codimension of *M*. Let $$\Omega = {\text {int}} (M+i \Gamma _M)$$. The set $$\overline{\Omega }$$ is the union of all translates of *M* in the directions spanned by the cone, $$i\Gamma _M$$. It is natural to define an $$H^p$$ function on $$\overline{\Omega } $$ to be a function, *f*, that is analytic on $$\Omega $$ and has a finite upper bound for all the $$L^p$$ norms of *f* on these translates of *M*. Similarly, there is an $$H^p_{CR} $$ theory for CR functions on lower-dimensional objects which have boundaries that contain *M* and emanate out from *M* in some subset of the directions which are spanned by $$i\Gamma _M$$. As an important case, suppose $$m=2$$. We define

$$\begin{aligned} M_1 = \{M+i v_2 V_2: v_2 \ge 0\} \ \ \text {and} \ \ M_2 = \{M+i v_1 V_1: v_1 \ge 0\}. \end{aligned}$$

An $H^p$ CR-function on $M_1$ or $M_2$ is one that has a uniform bound on all the $L^p$ norms of *f* over all translates of *M* in the direction of $iV_2$ or $iV_1$, respectively.

Our first main result (Theorem 3.3) is that an $H^p$ function on $\Omega$ has boundary values on *M* as an element in $CR^p(M)$. Conversely, an $L^p$ CR function on *M* extends to a unique element of $H^p(\overline{\Omega })$. This result is related to an earlier result of Boggess which has a more general hypothesis on *M* but a weaker conclusion [3].

Our results also apply to $M_1$ and $M_2$. A function in $H^p_{CR}(M_1)$ or $H^p_{CR}(M_2)$ has boundary values on *M* as an element of $CR^p(M)$. And conversely, an element of $CR^p(M)$ extends to a function in $H^p_{CR}(M_1)$ and a function in $H^p_{CR}(M_2)$. Our results include the analogous theorems for arbitrary codimension $m \ge 2$. Precise assumptions and definitions are given in Sects. 2 and 3.

As a related result, we prove a relationship between the Szegö kernel for *M* and the Bergman kernel for $\Omega ={\text {int}}(M+i \Gamma _M)$ (Theorems 4.2 and 6.1), again with the assumption that $\Gamma _M$ is closed and not equal to $\mathbb {R}^m$. For these results, we assume that $\Gamma _M$ is equal to the convex hull of a set of linearly independent vectors, $V_1, \dots , V_m \in \mathbb {R}^m$ (instead of the weaker assumption that $\Gamma _M$ is the convex hull of $V_1, \dots , V_N$ where $m \le N \le n$ as in Sects. 2 and 3). These results are the higher codimensional version of (2) from Nagel et al. [8] when *M* is a quadric CR manifold.

The authors would like to thank Alex Nagel for sharing the details of the integration by parts argument in [6, 8].

## The Levi Form Assumptions

As usual, let $\Gamma _M\subset \mathbb {R}^m$ be the convex hull of the image of the Levi form of $M \subset \mathbb {C}^n \times \mathbb {C}^m$ (i.e., the convex hull of the image of $z \mapsto \phi (z,z) \in \mathbb {R}^m$). Our geometric assumption on $\Gamma _M$ for the $H^p$ results given in the next section is as follows:

### Definition 2.1

A quadric $M \in \mathbb {C}^n \times \mathbb {C}^m$ is said to be *admissible* if there exists a set of spanning vectors $\{V_1, \dots , V_N \}$ for $\mathbb {R}^m$ whose convex hull equals $\Gamma _M$.

More explicitly, there exist vectors $Z_j \in \mathbb {C}^n$, $1 \le j \le N$ with $\phi (Z_j, Z_j)= V_j$ and

3 $$\begin{aligned} \Gamma _M = \left\{ \sum _{j=1}^N y_j \phi \left( Z_j, Z_j\right) = \sum _{j=1}^N y_jV_j : y_j \ge 0, \ 1 \le j \le N \right\} . \end{aligned}$$

### Remark 2.2

Since $\{V_1, \dots , V_N \}$ spans $\mathbb {R}^m$, note that $N \ge m$.

**Normal Form for** $\phi$. By adding some additional geometric conditions on $\Gamma _M$, we can obtain a normal form for $\phi$, which elucidates an important subclass of quadrics that satisfy our assumption (3). However, this normal form is not needed for the $H^p$ results of this paper.

### Definition 2.3

A spanning set of vectors $\{V_1,\dots , V_N\}$ whose convex hull is the polygonal cone $\Gamma \subset \mathbb {R}^m$ is said to be a *minimal generating set* if $\Gamma$ cannot be generated by any proper subset of $\{V_1, \dots , V_N \}$.

This definition is equivalent to stating that each ray through $V_j$ is not in the convex hull of the other rays through $V_k$, $1 \le k \not = j \le N$. If we also add the restriction that $\Gamma _M-\{0\}$ is contained in an open half-space in $\mathbb {R}^m$, then we get the following normal form for *M*.

### Proposition 2.4

Suppose *M* is admissible and suppose $\Gamma _M$ has a minimal generating set $\{V_1, \dots , V_N \}$ in $\mathbb {R}^m$ with $m \le N \le n$. If $\Gamma _M-\{0\}$ is contained in an open half space in $\mathbb {R}^m$, then there is a choice of coordinates for $\mathbb {C}^n \times \mathbb {C}^m$ such that

4 $$\begin{aligned} \phi (z,z) = \sum _{j=1}^N |z_j|^2 V_j. \end{aligned}$$

### Remark 2.5

Note that the assumption in (3) is invariant under complex linear changes of variables in $z \in \mathbb {C}^n$ but the normal form in (4) is not. For example, suppose $m=n=2$ and let $\phi _1(z,z) = |z_1|^2$ and $\ \phi _2(z,z) = |z_2|^2$; the complex linear change of variables given by $\hat{z}_1= z_1+z_2$, $\hat{z}_2 = z_2$ will result in a term of the form ${{\,\textrm{Re}\,}}\hat{z}_1 \overline{\hat{z}_2}$ in the new variables.

### Proof

Let $Z= \sum _{j=1}^N z_j Z_j$. Then we have

5 $$\begin{aligned} \phi (Z,Z) = \sum _{j=1}^N |z_j|^2 V_j + \sum _{1 \le j < k \le N} {{\,\textrm{Re}\,}}\{ z_j \bar{z}_k V_{j,k} \}, \ \ \text {with } m \le N \le n \end{aligned}$$

where, by assumption (3), $V_j=\phi (Z_j, Z_j)$, $1 \le j \le N$ and $V_{j,k} =2 \phi (Z_j, Z_k)$. The convex hull of $V_1, \dots , V_N$ equals $\Gamma _M$. However, note that the ${{\,\textrm{Re}\,}}V_{j,k}$ and ${{\,\textrm{Im}\,}}V_{j,k}$ a priori do not necessarily belong to $\Gamma _M$. We will show that since $\phi (Z,Z)$ belongs to $\Gamma _M$, $V_{j,k}$ must be zero.

By assumption, $\Gamma _M$ is contained in an open half space. Therefore each ray through $V_j$ is also contained in this half space. Furthermore, because the $V_j$ are a minimal generating set for $\Gamma _M$, each of these rays is not in the convex hull of the rays through the other $V_k$, $k \not = j$. This implies the following for each $V_j$: Let *L* be any open line segment in $\mathbb {R}^m$ which contains the point $V_j$ in its interior. The disconnected line segment, $L- \{V_j \}$, consists of two connected open line segments, which we will denote by $L_A$ and $L_B$. If *L* is not contained in the ray through $V_j$ from the origin, then because the $\{V_j; \ 1 \le j \le N\}$ form a minimal generating set, either $L_A \cap \Gamma _M= \emptyset$ or $L_B \cap \Gamma _M = \emptyset$ (or both).

Now fix any $j \not =k$ and let $z_j=1$ and $z_k = t \in \mathbb {R}$ in (5), and set all other $z_\ell =0$, for $\ell \not = j,k$. The resulting value of $\phi (Z,Z)$ is $\tilde{\phi }(t):=V_j+t {{\,\textrm{Re}\,}}V_{j,k} +t^2V_k$. This quantity must belong to $\Gamma _M$ for all $t \in \mathbb {R}$. Consider the line segment *L* parameterized by $t \mapsto V_j+t {{\,\textrm{Re}\,}}V_{j,k}$ for |*t*| small. This line segment contains $V_j$ (when $t=0$). By the previous paragraph, if ${{\,\textrm{Re}\,}}V_{j,k}$ is not a multiple of $V_j$, then either $L_A \cap \Gamma _M=\emptyset$ or $L_B \cap \Gamma _M= \emptyset$. Since the curve parameterized by $\tilde{\phi }(t)= V_j+t {{\,\textrm{Re}\,}}V_{j,k}+t^2 V_k$ is tangent to *L* at the point $V_j$, we conclude that for some $\varepsilon >0$, either the curve parameterized by $\tilde{\phi }(t)$ for $0<t< \varepsilon$ or $-\varepsilon< t< 0$ (or possibly both) has no intersection with $\Gamma _M$. This contradicts the fact that $\tilde{\phi }(t)$ belongs to $\Gamma _M$ for all *t*. We conclude ${{\,\textrm{Re}\,}}V_{j,k}$ must be a multiple of $V_j$.

Now repeat this analysis with the roles of *j* and *k* reversed, i.e., set $z_j=t \in \mathbb {R}$ and $z_k = 1$ and set all other $z_\ell =0$, for $\ell \not = j,k$. One concludes that ${{\,\textrm{Re}\,}}V_{j,k}$ must also be a multiple of $V_k$. Since ${{\,\textrm{Re}\,}}V_{j,k}$ cannot be a nonzero multiple of both $V_j$ and $V_k$, we conclude that ${{\,\textrm{Re}\,}}V_{j,k}$ must be zero.

Repeating the above arguments with $z_j=1$ and $z_k = it \in \mathbb {R}$ in (5), and all other $z_\ell =0$, for $\ell \not = j,k$, we conclude that ${{\,\textrm{Im}\,}}V_{j,k} =0$. Thus, the description of $\phi$ in the new coordinates, $Z= \sum _{j=1}^N z_j Z_j$ satisfies (4), as desired. $\square$

### Remark 2.6

The following two examples show the necessity of the hypothesis on $\Gamma _M$ being contained in an open half space.

- Consider $$M= \{(z, w) \in \mathbb {C}^3 \times \mathbb {C}^2: {{\,\textrm{Im}\,}}w= \phi (z,z)\}$$ with
  $$\begin{aligned} \phi _1(z,z) = |z_1|^2- |z_2|^2; \ \ \phi _2(z,z) = {{\,\textrm{Re}\,}}z_1 \bar{z}_2-|z_3|^2. \end{aligned}$$
  $$\Gamma _M$$ is all of $$\mathbb {R}^2$$ and is generated by the vectors $$V_1= \phi (1,0,0)$$, $$V_2= \phi (1,2,0)$$, and $$V_3= \phi (1,2,2)$$. Any complex linear change of variables in *z* designed to arrange the normal form in (4) would have to leave $$z_3$$ alone (in view of the lone $$|z_3|^2 $$ term). This follows by setting $$z_3=0$$. Then, any complex linear change of coordinates in $$\mathbb {C}^2$$ that eliminates the $${{\,\textrm{Re}\,}}z_1 \bar{z}_2$$ term in $$\phi _2$$ and replaces it by a linear combination of $$|z_1|^2$$ and $$|z_2|^2$$ is not possible (this follows from Theorem 2 in Sect. 7 in [2]).
- Consider $$M= \{(z, w) \in \mathbb {C}^4 \times \mathbb {C}^3; \ {{\,\textrm{Im}\,}}w= \phi (z,z)\}$$ with
  $$\begin{aligned} \phi _1(z,z) = |z_1|^2- |z_2|^2: \ \phi _2(z,z) = {{\,\textrm{Re}\,}}z_1 \bar{z}_2-|z_3|^2; \ \ \phi _3(z,z) = |z_4|^2. \end{aligned}$$
  $$\Gamma _M$$ is the closed half space $$\{ {{\,\textrm{Im}\,}}w_3 \ge 0 \}$$ and is generated by the vectors $$V_1= \phi (1,0,0,0)$$, $$V_2= \phi (1,2,0,0)$$, $$V_3= \phi (1,2,2,0)$$, and $$V_4 = \phi (0,0,0,1)$$. Any complex linear change of variables in *z* designed to arrange the normal form in (4) would have to leave $$z_3$$ and $$z_4$$ alone (in view of the lone $$|z_3|^2 $$ and $$|z_4|^2$$ terms). Any change of variables in $$z_1$$ and $$z_2$$ designed to eliminate the $${{\,\textrm{Re}\,}}z_1 \bar{z}_2$$ term is not possible as in the previous example.

## $$H^p $$ Results for Quadrics

We assume *M* is admissible (as defined in Definition 2.1) and that $\Gamma _M$ satisfies (3).

### Definition 3.1

Let $I=\{1 \le i_1< i_2< \dots < i_k \le N\}$ be an index of length $|I|=k$, $0 \le k \le N$; define $I' = \{1, \dots N\} - I$, an index of length $N-k$. Define

$$\begin{aligned} M_I = \Big \{(z, t+i(\phi (z,z) +v_{I'})); v_{I'} = \sum _{j' \in I'} v_{j'} V_{j'}, \ v_{j'} \ge 0 \Big \}. \end{aligned}$$

Note that $\dim _\mathbb {R}(M_I) = \dim _\mathbb {R}(M) +|I'|=\dim _\mathbb {R}(M) +N-|I|$ if the vectors $\{V_j; \ j \in I'\}$ are linearly independent. In this case, if $|I'|>0$, then $M_I$ is a CR manifold with boundary that contains *M*. The CR structure is comprised by the CR vector fields of *M* translated in the directions given by the $iV_j, \ j \in I'$ together with the vector fields $(V_j-iJV_j)$, $j \in I'$ (and their conjugates). If the vectors, $V_j$, are not linearly independent, then the same statement on the CR structure is true by restricting to a subset of the $V_j$ which form a basis for the vector space spanned by the $V_j, \ j \in I'$.

Here are a few important special cases: $$M_\emptyset =M+i \Gamma _M = \overline{\Omega }$$ and $$ M_{1, \dots , N}= M$$. Also, if the rank of the space spanned by $$\{V_j; \ j \in I'\}$$ in $$\mathbb {R}^m$$ is *m*, then $$M_I $$ is a subset of $$\overline{\Omega }$$ with nonempty interior. As another example, suppose $$m=N=2$$ and $$V_j = E_j$$, $$j=1,2$$, a standard basis vector. Then

$$\begin{aligned} M_1&= \{(z, t+i \phi (z,z) +(0, 0,iv_2)) \in \mathbb {C}^n \times \mathbb {C}\times \mathbb {C}; \ v_2 \ge 0 \} \\ M_2&= \{ (z, t+i \phi (z,z)+(0, iv_1, 0)) \in \mathbb {C}^n \times \mathbb {C}\times \mathbb {C}; \ v_1 \ge 0 \}. \end{aligned}$$

We define the following $H^p$ spaces of functions on $\Omega$ and the $M_I$.

### Definition 3.2

Assume $\Gamma _M$ is closed and is the convex hull of a set of spanning vectors $V_1, \dots , V_N$ for $\mathbb {R}^m$ as stated in the Assumption (3).

- Let $CR^p(M)$ be the space of all CR functions (or distributions) on *M* that are in $L^p(M)$ with the norm:
  $$\begin{aligned} \Vert f\Vert ^p_{L^p(M)}:= \int _{\alpha ' \in M} |f(\alpha ')|^p \, d \alpha ' = \int _{(z,x) \in \mathbb {C}^n\times \mathbb {R}^m} |f(z, x+i\phi (z,z))|^p \, d \alpha ' \end{aligned}$$
  where $d \alpha ' = dv(z)\,dx$ = the volume form for $\mathbb {C}^n \times \mathbb {R}^m$.
- Let $H^p(\overline{\Omega })$ be the space of holomorphic functions *F* in $\Omega$ satisfying
  $$\begin{aligned} \Vert F\Vert _{H^p (\bar{\Omega })}:=\sup _{V \in \Gamma _M} \int _{\alpha ' \in M} |F(\alpha ' +iV)|^p \, d \alpha ' < \infty . \end{aligned}$$
- For any index *I*, let $$H^p_{CR}(M_I) $$ be the space of all CR functions $$F_I$$ on $$M_I$$ satisfying
  6 $$\begin{aligned} \Vert F_I\Vert _{H^p_{CR}(M_I)}:=\sup _{y_{I'}} \int _{\alpha ' \in M} |F_I(\alpha ' +iy_{I'})|^p \, d \alpha ' < \infty . \end{aligned}$$
  Here, $$y_{I'} = \sum _{j' \in I'} y_{j'} V_{j'}; \ y_{j'} \ge 0$$.

We want to think of $CR^p(M)$ as boundary values of $H^p$ functions on $\Omega$ and as boundary values of functions in $H^p_{CR} (M_I)$, for any index *I*. Our main theorem (Theorem 3.3) establishes the precise sense in which these intuitions are correct.

### Theorem 3.3

Suppose that *M* is a codimension *m* admissible quadric in $\mathbb {C}^{n+m}$ defined by (1) ($\Gamma _M$ satisfies (3)). Let $1 \le p < \infty$ and suppose that $f \in CR^p(M)$. Let $I \subset \{1, \dots , N \}$ be any increasing index and let $I' = \{1, \dots , N\}-I$. Then there exists a unique $F_I \in H^p_{CR}(M_I)$ that extends *f* in the sense that

7 $$\begin{aligned} \lim _{y_{I'} \rightarrow 0} \int _{\alpha ' \in M} |F_I(\alpha '+iy_{I'})-f(\alpha ')|^p\, d\alpha ' =0, \quad \text {if } \ 1 \le p < \infty \end{aligned}$$

and

8 $$\begin{aligned} \Vert F_I\Vert _{H^p_{CR}(M_{I})} = \Vert f\Vert _{L^p(M)}. \end{aligned}$$

Here, $y_{I'} = \sum _{j' \in I'} y_{j'} V_{j'}; \ y_{j'} \ge 0$. Conversely, let $I \subset \{1, \dots , N \}$ be any increasing index. If $F_I \in H^p_{CR}(M_I)$, then there exists a unique $f \in CR^p(M)$ such that (7) and (8) hold.

### Remark 3.4

When $I= \emptyset$, $M_I= \overline{\Omega }$. In this case, (7) reads

9 $$\begin{aligned} \lim _{y \rightarrow 0, \ y \in \Gamma _M} \int _{\alpha ' \in M} |F(\alpha '+iy)-f(\alpha ')|^p\, d\alpha ' =0. \end{aligned}$$

This special case will be stated and proved below (see Theorem 3.9) since we will need it for the proof of Theorem 3.3.

As another special case of Theorem 3.3, with $m=2$ and $I=\{1\}$ (and so, $I' =\{2\}$), we obtain the following result.

### Theorem 3.5

Suppose that *M* is a codimension two quadric in $\mathbb {C}^{n+2}$ defined by (1) satisfying the assumption given in (3) with $V_j=E_j, \ j=1,2$, the standard basis vectors in $\mathbb {R}^2$, (so $\Gamma _M = \{y=(y_1, y_2); \ y_j \ge 0, \ j=1,2 \}$). Let $1 \le p < \infty$ and suppose that $f \in CR^p(M)$. Then there exists a unique $\tilde{f} \in H^p_{CR}(M_1)$ that extends *f* in the sense that

10 $$\begin{aligned} \lim _{y_2 \rightarrow 0^+ } \int _{\alpha ' \in M} |\tilde{f}(\alpha ' +iy_2 E_2)-f(\alpha ')|^p\, d\alpha ' =0, \quad \text {if }1 \le p < \infty \end{aligned}$$

and

11 $$\begin{aligned} \Vert \tilde{f}\Vert _{H^p_{CR}(M_1)} = \Vert f\Vert _{L^p(M)}. \end{aligned}$$

Conversely, if $\tilde{f} \in H^p_{CR}(M_1)$, then there exists $f \in CR^p(M)$ such that (10) and (11) hold.

### Remark 3.6

Of course, the above theorem could have been stated just as easily for $M_2$.

We now prove Theorem 3.3. For some of the steps in the proof, we will assume that $m=2$ and prove Theorem 3.5, which avoids some of the complexity of notation of the general case. The ideas in the proof of the general case are the same as those in the proof of Theorem 3.5. We focus first on the converse case where the starting point is a given $\tilde{f} \in H^p_{CR}(M_1)$. Our first goal is to show that $\tilde{f}$ has boundary values on *M* as an element of $CR^p(M)$. We accomplish this in Steps 1 and 2 below.

### Proof of Theorem 3.5

**Step 1.** Suppose $\tilde{f} \in H^p_{CR} (M_1)$. For $\varepsilon >0$, let

$$\begin{aligned} f_\varepsilon \left( \alpha '\right) = \tilde{f}(\alpha '+i\varepsilon E_2), \ \ \text {for } \alpha ' \in M. \end{aligned}$$

The assumption in (6) implies there is a uniform bound on the $L^p(M)$-norms of $f_\varepsilon$. Because the unit ball in $L^p(M)$ has compact closure in the weak* topology, for $1 \le p < \infty$ there is an $f \in CR^p(M)$ and a sequence $\varepsilon _j \rightarrow 0$ such that

$$\begin{aligned} \langle f_{\varepsilon _j}, \psi \rangle \rightarrow \langle f, \psi \rangle , \ \ \text {for each } \psi \in L^q(M), \frac{1}{p}+\frac{1}{q}=1. \end{aligned}$$

**Step 2.** Next, we show that $f_\varepsilon \rightarrow f$, as $\varepsilon \rightarrow 0$, as distributions on *M*. Furthermore, we show that if $f=0$, then $\tilde{f}=0$. First notice that $M_1$ is foliated with copies of the upper half space in $\mathbb {C}$, denoted $H^+$, since we can write a point of $M_1$ as

12 $$\begin{aligned} p&= (z, x+i \phi (z,z)) + (0,0+iv_2E_2 ), \ \ z \in \mathbb {C}^n, \ \ x =(x_1, x_2) \in \mathbb {R}^2, \ \ v_2 >0 \nonumber \\&=(z, x_1+i \phi _1(z,z), i \phi _2(z,z)) + (0,0, w_2) \in \mathbb {C}^n \times \mathbb {C}\times H^+ \end{aligned}$$

where $w_2 = x_2+i v_2$. In what follows, we identify the point $p \in M_1$ with $(z, x_1, w_2)$. Note that *p* belongs to *M* if and only if $v_2=0$. Let

$$\begin{aligned} \bar{L} = \frac{\partial }{\partial \bar{w}_2} = \frac{1}{2}\Big ( \frac{\partial }{\partial x_2} +i \frac{\partial }{\partial v_2}\Big ) \end{aligned}$$

and note that $\bar{L}$ belongs to $T^{0,1}(M_1)$. In particular, if *f* is a CR distribution on $M_1$, then $\bar{L} f=0$ on $M_1$, i.e., *f* is holomorphic in $w_2=x_2+iv_2$ for $w_2 \in H^+$.

In our context, $\tilde{f} \in H^p_{CR}(M_1)$, which easily implies $\tilde{f}$ is locally integrable on $M_1$ as well as $\bar{L}\tilde{f} =0$ on $M_1$. Therefore, Step 2 follows from the theorems in Chapter VI of [1]. For completeness, we outline the ideas in [1] as adapted to our setting to show that $f_\varepsilon \rightarrow f$ as distributions.

Suppose $\tilde{f}: M_1 \rightarrow \mathbb {C}$. We use (12) to identify a point $p \in M_1$ with $(z, x_1, w_2)$ where $w_2 = x_2+iv_2 \in H^+ \subset \mathbb {C}$. We define

$$\begin{aligned} \tilde{f}_{z,x_1}(w_2) = \tilde{f}((z, x_1+i \phi _1(z,z), i \phi _2(z,z)) + (0,0,w_2)). \end{aligned}$$

We fix a point $p_0 \in M$ and let $U_0$ be a neighborhood of $p_0$ in $M_1$ of the form

$$\begin{aligned} U_0= U_{n+1} \times \{a<x_2< b\} \times \{0<v_2<c \} \end{aligned}$$

where $U_{n+1}$ is a bounded open set in $\mathbb {C}^n \times \mathbb {R}$. We now use the ideas in [1] to prove the following lemma. $\square$

### Lemma 3.7

Suppose $\tilde{f}_{z,x_1}(w_2)$ is holomorphic in $w_2=x_2+iv_2$ for $\{a<x_2< b\} \times \{0<v_2<c \}$ and $(z,x_1) \in U_{n+1}$. Furthermore, suppose that there exists an integer $\ell \ge 0$ such that the function

13 $$\begin{aligned} (z,x_1, w_2) \mapsto \tilde{f}_{z,x_1}(w_2)v_2^\ell , \ \ (z,x_1, w_2) \in U_0 \end{aligned}$$

is integrable in $U_0$. Then $\lim _{v_2 \rightarrow 0^+} \tilde{f}_{z,x_1}(x_2+iv_2)$ exists as a distribution of order $\ell +1$ on the set $\{(z,x_1) \in U_{n+1} \} \times \{a<x_2<b\}$, which corresponds to an open subset of *M* through the identification in (12). Moreover, if this limit is zero (in the sense of distributions), then $\tilde{f}_{z,x_1}(w_2) = 0$ for $w_2=x_2+iv_2$ in $\{a<x_2< b\} \times \{0<v_2<c \}$ and $(z,x_1) \in U_{n+1}$.

### Remark 3.8

In our case with $\tilde{f} \in H^p_{CR}(M_1)$, the integrability assumption in (13) is satisfied with $\ell =0$. Once this lemma has been established, we conclude that $\lim _{\varepsilon \rightarrow 0} f_\varepsilon =f$, as distributions, in view of Step 1.

### Proof of Lemma 3.7

Let $\psi$ be a $C_0^\infty$ function with support contained in $U_{n+1} \times \{a<x_2<b\}$. We will set $v_2=\varepsilon >0$ and show

14 $$\begin{aligned} \lim _{\varepsilon \rightarrow 0^+} \int _{U_{n+1}} \int _a^b \tilde{f}_{z,x_1}(x_2+i\varepsilon ) \psi (z,x_1, x_2) \, dx_2 \, dv(z) \, dx_1 \end{aligned}$$

exists.

Let $c>0$ be specified as in the statement of the lemma. Given $\psi$ as above, we construct a smooth $\psi _\ell : U_{n+1} \times \{a<x_2<b\} \times \{ 0 \le v_2 <c \} \rightarrow \mathbb {C}$ with the following properties:

A. $$\psi _\ell (z, x_1, x_2+i(v_2 =0)) = \psi (z, x_1, x_2)$$ for $$(z, x_1) \in U_{n+1}$$ and $$ a< x_2 < b$$
B. $$|\bar{L} \psi _\ell (z, x_1, w_2) | \le C_{m+1}\, v_2^\ell $$ for $$(z, x_1) \in U_{n+1}$$ and $$w_2 = x_2+iv_2 $$ with $$a< x_2 < b$$, $$ 0 \le v_2 \le c$$ where $$C_{\ell +1} $$ is a constant that depends only on the supremum of the $$x_2$$ derivatives of $$\psi $$ through order $$\ell +1$$.

In fact, it is an easy exercise to show

$$\begin{aligned} \psi _\ell (z, x_1, w_2):= \sum _{k=0}^\ell \frac{\partial ^k \psi (z, x_1, x_2)}{\partial x_2^k} \frac{(iv_2)^k}{k!} \end{aligned}$$

satisfies both properties.

Now for any $0< \varepsilon <c$, use Green’s Theorem in the rectangle $a\le x_2 \le b$, $\varepsilon \le v_2 \le c$ in the $w_2$-plane to establish

15 $$\begin{aligned}&\int _{(z,x_1) \in U_{n+1}} \int _a^b \tilde{f}(z,x_1, x_2 {+i\varepsilon }) \psi _m(z,x_1, x_2+i \varepsilon ) \, dx_2 \, dv(z) \, dx_1 \end{aligned}$$

16 $$\begin{aligned}&-\int _{(z,x_1) \in U_{n+1}} \int _a^b \tilde{f}(z,x_1, x_2 +ic) \psi _m(z,x_1, x_2+ic) \, dx_2 dv(z) \, dx_1 \nonumber \\&={2i}\int _{(z,x_1) \in U_{n+1}} \int _a^b \int _\varepsilon ^c \bar{L} (\tilde{f} \psi _m ) (z, x_1, w_2) \, d v_2 \, dx_2\, dv(z) \, dx_1 \nonumber \\&= {2i} \int _{(z,x_1) \in U_{n+1}} \int _a^b \int _\varepsilon ^c \tilde{f}(z, x_1, w_2) \, \bar{L}\psi _m (z, x_2, w_2) \, dv_2 \, dx_2 \, dv(z) \, dx_1 \ \ \text {since }\bar{L} {\tilde{f}} =0 . \end{aligned}$$

In view of property B above for $\psi _m$ and the integrability hypothesis in (13), we can let $\varepsilon \rightarrow 0^+$ in the last integral on the right and conclude that the limit as $\varepsilon \rightarrow 0^+$ of the expression in (15) exists, as desired. This concludes the proof of the existence of the limit in (14).

Note: The above arguments establishing the first part of Lemma 3.7 can be generalized to handle the $M_I$ in Theorem 3.3. Here is an outline. First note that it suffices to consider the case when $N=m$ and the vectors $V_{i}$, $1 \le i \le m$ are linearly independent. Indeed for the general case, $M_I$ can be written as a finite union of $M_J$ where $N=m$ and the $V_{j}$ associated with *J* and $J'$ are linearly independent and are subsets of of the $V_{i_j}$ and $V_{i'_j}$ associated with *I* and $I'$, respectively. Thus, we assume $N=m$ and the vectors $V_i, \ 1 \le i \le m$ are linearly independent.

Let $k''$ be the dimension of the space spanned by the $\{V_j; \ j \in I'\}$ (note $k'' \le m$), and use a complex linear change of variables so that $I=\{1, \dots , m-k'' \}$ and $I' =\{m-k''+1, \dots , m\}$ and $V_i=E_i$ for $1 \le i \le m$. Then let

$$\begin{aligned} \psi _\ell \left( z, x', x''+iv''\right) = \sum _{|\alpha | \le \ell } \frac{ \partial ^{|\alpha | } \psi (z,x^I, x^{I'})}{\partial x^{\alpha } } \frac{(iv_{I'})^{\alpha }}{\alpha !}. \end{aligned}$$

In the sum, the multi-index $\alpha = (\alpha _{m-k''+1}, \dots , \alpha _{m})$ runs over all indices with $|\alpha | \le \ell$. One can show that for each $m-k''+1 \le j \le m$

17 $$\begin{aligned} |\bar{L}_{j} \psi _\ell \left( z, x', x''+iv''\right) | \le C_{\ell }|v''|^\ell . \end{aligned}$$

The analogous calculations for (15) and (16) involve Stokes’ Theorem over a surface that connects $\{(z,x', x''+i\varepsilon ''); z \in \mathbb {C}^m, \ x' \in \mathbb {R}^{m-k''}, \ x'' \in \mathbb {R}^{k''} \}$ to $\{(z,x', x''+ic''); z \in \mathbb {C}^m, \ x' \in \mathbb {R}^{m-k''}, \ x'' \in \mathbb {R}^{k''} \}$ where $c''$ is a fixed point in $\mathbb {R}_+^{k''}$ and $\varepsilon '' \in \mathbb {R}_+^{k''}$ which is allowed to get arbitrarily small. Details are left to the reader.

To show uniqueness, we assume the limit in (14) is zero for all smooth functions $\psi$ with support contained in $U_{n+1} \times \{a<x_2<b\}$. We wish to show $\tilde{f}_{z,x_1}(x_2+iv_2) =0$ for $v_2>0$. Let $\psi$ be given and define

18 $$\begin{aligned} \hat{F}_\psi (w_2)=&\int _{(z,x_1) \in U_{n+1}} \int _{x_2=a}^b \tilde{f}_{z,x_1} (x_2 + w_2) \psi (z,x_1, x_2) \, d x_2 \, dv(z) \, dx_1 \text {where }\nonumber \\&w_2 = u_2+iv_2 \in H^+. \end{aligned}$$

We restrict $$u_2 = {{\,\textrm{Re}\,}}{w_2} $$ so that $$|u_2| < \delta $$ where $$\delta $$ is chosen (depending on $$\psi $$) so that the $$x_2$$-support of $$\psi $$ is contained in $$a + \delta< x_2 < b-\delta $$. With this restriction, $$\hat{F}_\psi (w_2)$$ is analytic in the region $$|{{\,\textrm{Re}\,}}w_2|<\delta $$ and $$0< {{\,\textrm{Im}\,}}w_2 < c$$. Furthermore, an easy change of variables: $$\hat{x}_2 = x_2+u_2 = x_2 + {{\,\textrm{Re}\,}}w_2$$ in the above $$x_2 $$-integral yields

$$\begin{aligned} \hat{F}_\psi (w_2)=&\int _{(z,x_1) \in U_{n+1}} \int _{\hat{x}_2=a}^b \tilde{f}_{z,x_1} (\hat{x}_2 +i v_2) \psi (z,x_1, \hat{x}_2-u_2) \, d \hat{x_2} \, dv(z) \, dx_1 \text {where }\\&w_2=u_2+iv_2. \end{aligned}$$

Note that the $z, x_1, \hat{x}_2$-support of $\psi (z,x_1, \hat{x}_2-u_2)$ is still contained in $U_{n+1} \times \{a< \hat{x}_2<b\}$ provided $|u_2| < \delta$. Therefore by our assumption,

$$\begin{aligned} \lim _{v_2 \rightarrow 0^+} \hat{F}_\psi (u_2+iv_2) = 0 \ \ \text {for }|u_2| < \delta . \end{aligned}$$

By the Schwarz Reflection Principle, $\hat{F}_\psi (w_2)$ analytically extends to $|u_2|< \delta , \ -c< v_2 <0$, and furthermore, $\hat{F}_{\psi }(u_2+i0) =0$. By uniqueness, $\hat{F}_\psi (w_2) = 0$ for all $w_2=u_2+iv_2$ in the strip $|u_2|< \delta$ and $|v_2|<c$. In particular, $\hat{F}_\psi (iv_2)=0$ for all $|v_2|<c$ and so the integral in (18) is zero for every $\psi$ with compact support in $U_{n+1} \times \{ a<x_2< b \}$. We conclude that $\tilde{f}_{z,x_1}(x_2+iv_2)= 0$ for $0< v_2 < c$, for any arbitrary $c>0$. This uniqueness argument can be modified for the general case. $\square$

This concludes the proof of uniqueness and thus Step 2 is complete.

**Step 3.** The next step is to extend $f \in CR^p(M)$ (constructed in Step 1) to an element *F* in $H^p(\overline{\Omega })$, where $\Omega = {\text {int}} ( M+i \Gamma _M)$. We will prove the following key theorem, which is stated and proved in full generality for admissible quadrics.

### Theorem 3.9

Let $M \subset \mathbb {C}^{n+m}$ be any admissible quadric. Let $\Omega = {\text {int}} (M+i \Gamma _M )$. Fix any $1 \le p < \infty$ and suppose $f \in CR^p(M)$. Then there exists a unique $F \in H^p (\overline{\Omega })$ that extends *f* in the sense that

19 $$\begin{aligned} \lim _{y \in \Gamma _M, \ y\rightarrow 0} \int _{\alpha ' \in M} |F(\alpha '+iy)-f(\alpha ')|^p\, d\alpha ' =0. \end{aligned}$$

Furthermore,

20 $$\begin{aligned} \Vert F\Vert _{H^p(\bar{\Omega }) } = \Vert f\Vert _{L^p(M)} \end{aligned}$$

A related theorem was originally proved in [3] where the quadric *M* satisfied more general hypotheses but the conclusion in (19) was limited to *y* belonging to a smaller subcone within $\Gamma _M$. Due to this restriction on *y*, the $H^p$ extension of *f* given in [3] cannot be restricted to $M_I$.

To prove Theorem 3.9, we will need the following approximation theorem, as applied to quadrics, given in [3].

### Theorem 3.10

[Theorem 2, [3]] Suppose $$M \subset \mathbb {C}^{n+m}$$ is a quadric submanifold as given in (1) and suppose $$1 \le p < \infty $$. Let *f* be an element of $$ CR^p(M)$$. Then there is a sequence of entire functions $$F_k$$ such that on each compact set $$K \subset M$$, $$F_k \rightarrow f $$ in $$L^p(K)$$, as $$k \rightarrow \infty $$.

We will use the above theorem together with the following family of analytic discs to find our $H^p$ extension of $f \in CR^p(M)$. Let $\Delta$ be the closed unit disc in $\mathbb {C}$. Let $Z_j$ and $V_j = \phi (Z_j, Z_j)$, for $1 \le j \le N$, as in the relaxed assumption (3). For $z \in \mathbb {C}^n$ and $s=(s_1, \dots , s_N ) \in \mathbb {R}^N$ with $s_j \ge 0, \ 1 \le j \le N$, define $Z_{s,z}: \Delta \rightarrow \mathbb {C}^n$ to be the analytic disc

21 $$\begin{aligned} Z_{s,z} (\zeta ) := z+Z^0_s (\zeta ) \ \ \text {where } Z^0_s(\zeta )=\sum _{j=1}^N s_j Z_j \zeta ^j. \end{aligned}$$

For each $x \in \mathbb {R}^m$, define the analytic discs $G_{s, z}: \Delta \rightarrow \mathbb {C}^m$ and $A_{x,s,z}: \Delta \rightarrow \mathbb {C}^{n} \times \mathbb {C}^m$ by

22 $$\begin{aligned} G_{s,z}(\zeta ) =&i \left[ \phi (z,z) + \sum _{j=1}^N s_j^2 V_j \right] \nonumber \\&+ 2i \left[ \sum _{j=1}^N s_j \phi (Z_j, z) \zeta ^j + \sum _{1 \le j<k\le N} s_js_k \phi (Z_k, Z_j) \zeta ^{k-j} \right] \end{aligned}$$

23 $$\begin{aligned} A_{x,s,z}(\zeta )&= (z+Z^0_{s} (\zeta ), \ x+ G_{s,z} (\zeta )). \end{aligned}$$

Using the identity $V_j = \phi (Z_j, Z_j)$, it is a straightforward calculation that

24 $$\begin{aligned} {{\,\textrm{Im}\,}}\{x+G_{s,z}( \zeta )\} = \phi \big (z+Z^0_{s} (\zeta ),z+ Z^0_{s} (\zeta )\big ) \ \ \text {for }|\zeta | = 1 \end{aligned}$$

and so the boundary of the disc $A_{x,s,z}$ is contained in *M*. Furthermore, the center of the analytic disc, *A*, is

25 $$\begin{aligned} A_{x,s,z}( \zeta =0) =\left( z, x+i \phi (z,z) +i \sum _{j=1}^N s_j^2 V_j \right) . \end{aligned}$$

The right side parameterizes $M+i \Gamma _M$ as $z \in \mathbb {C}^n, \ x \in \mathbb {R}^m$ and $s_j \ge 0$, $1 \le j \le N$ vary. Also note that $A_{x,s,z}$ depends continuously on the parameters $x \in \mathbb {R}^m, s \in \mathbb {R}_+^N, \ z \in \mathbb {C}^n$ where $\mathbb {R}_+ = \{ s \in \mathbb {R}; \ s \ge 0 \}$. Furthermore, we have the following proposition which states that any compact set in $M+i\Gamma _M$ can be swept out by the centers of the analytic discs, $A_{x,s,z}$, whose boundaries are then contained in a compact set in *M*. More precisely:

### Proposition 3.11

Given any compact set $$K_1 $$ in $$\mathbb {C}^{n+m}$$, there are compact sets $$P_2 \subset \mathbb {R}^m \times \mathbb {R}_+^N \times \mathbb {C}^n $$ and $$K_2 \subset \mathbb {C}^{n+m}$$ such that the image of the map

$$\begin{aligned} (x,s, z) \in P_2 \mapsto A_{x,s, z}(\zeta =0) \end{aligned}$$

contains $K_1 \cap \{M+i\Gamma _M\}$ and the set of boundaries

$$\begin{aligned} \big \{A_{x,s,z}(\zeta ); \ |\zeta |=1, \ (x,s,z) \in P_2 \big \} \end{aligned}$$

is contained in $K_2 \cap M$.

The proposition is easy to prove. The existence of $P_2$ in the parameter set is evident from the formula for the center of *A* given in (25). The existence of $K_2$ then follows from the fact that $A_{x,s,z}(\zeta )$ depends continuously on its parameters and $\zeta$.

### Proof of Theorem 3.9

Suppose $q = (z, x+i \phi (z,z) +iy )$ is a given point in $\Omega$ (i.e., $y=\sum _{j=1}^N y_j V_j \in \Gamma$ with $y_j \ge 0$). Suppose $K_1 \subset \mathbb {C}^n \times \mathbb {C}^m$ is a compact set in $\Omega$ whose interior contains *q*. Let $P_2$ and $K_2$ be the compact sets given in Proposition 3.11. Now let $\{F_k\}$ be the sequence of entire functions given in Theorem 3.10, which converges to *f* in $L^p(K_2 \cap M)$. Let *U* be an open neighborhood in $\mathbb {C}^{n+m}$ of *q* such that $\bar{U} \subset \text {int} (K_1)$. We claim $F_k$ is uniformly convergent on *U*. Elementary Cauchy estimates for analytic functions imply

$$\begin{aligned} \sup _{U} |F_j - F_k| \le C \Vert F_j-F_k\Vert _{L^p(K_1)} \ \ j, k \ge 1 \end{aligned}$$

where *C* is a uniform constant depending only on *U* and $K_1$. Since the set of centers of the analytic discs, $A_{x,s, z}(\zeta =0)$ for $(x,s, z) \in P_2$ contains $K_1$, we can use subaveraging estimates as follows:

26 $$\begin{aligned}&\sup _{U} |F_j-F_k|^p \le C \int _{(x,s, z) \in P_2} |F_j(A_{x,s, z}(\zeta =0))-F_k(A_{x,s,z}(\zeta =0))|^p \, dx \, ds \, dv(z) \end{aligned}$$

27 $$\begin{aligned}&\le \frac{C}{2 \pi } \int _{(x,s, z) \in P_2} \int _{t=0}^{2 \pi } |F_j(A_{x,s, z}(e^{it}))-F_k(A_{x,s, z}(e^{it}))|^p \, dt \, dx \, ds \, dv(z) \end{aligned}$$

where *dv*(*z*) is the volume form on $\mathbb {C}^n$. Note that $dx \, dv(z)$ is Haar measure on *M*. Also, the boundary of the analytic discs, $A_{x,s, z} ( \cdot )$ for $(x,s, z) \in P_2$, are contained in the compact set $K_2 \cap M$.

To perform the (*z*, *x*)-integration in (27), note the formulas (23) and (22) and then make the change of variables

28 $$\begin{aligned} \hat{z}&= z+ Z^0_{s}( e^{it}) \ \ \text {and} \ \ \hat{x} = {{\,\textrm{Re}\,}}\left\{ x+G_{s,z}(e^{it}) \right\} = x- {{\,\textrm{Im}\,}}\left\{ G^0_{s,z}( e^{it}) \right\} \ \ \text {where} \end{aligned}$$

29 $$\begin{aligned} G^0_{s,z} (\zeta )&=2 \left[ \sum _{j=1}^N s_j \phi (Z_j, z) \zeta ^j + \sum _{1 \le j<k\le N} s_js_k \phi (Z_k, Z_j) \zeta ^{k-j} \right] \end{aligned}$$

Note that $z+Z^0_{s}( e^{it})$ and ${{\,\textrm{Im}\,}}\{G^0_{s,z}( e^{it})\}$ are independent of *x* and so the Jacobian of this change of variables is 1, i.e., $dx \, dv(z) \, ds \, dt = d \hat{x} \, d v(\hat{z}) \, ds \, dt$. So the (*z*, *x*) integral in (27) becomes the $(\hat{z}, \hat{x})$ integral, which is bounded by $\Vert F_j- F_k\Vert ^p_{L^p(K_2 \cap M)}$. The same is true after integrating out *t* and *s* within the parameter set in (27); we conclude

$$\begin{aligned} \sup _{U} |F_j-F_k| \le C \Vert F_j - F_k\Vert _{L^p(K_2 \cap M)}. \end{aligned}$$

Since $F_j \rightarrow f$ in $L^p (K_2 \cap M)$, the above inequality shows that $F_j$ is uniformly Cauchy on $U\subset K_1$. Since *U* contains *q* and *q* was arbitrarily chosen in $\Omega$, we conclude that $F_j$ converges to an analytic function *F* on $\Omega$.

Next, we show $\Vert F\Vert _{H^p(\overline{\Omega })} \le \Vert f\Vert _{L^p(M)}$. Take any $q = (z, x+i \phi (z,z) +iy) \in M +i \Gamma _M$. Recall that $y \in \Gamma _M$ can be expressed as $y = \sum _{j=1}^N y_j V_j$ with $y_j \ge 0$. Let $s= \sqrt{y} = \big ( \sqrt{y_1}, \dots , \sqrt{y_N} \big ) \in \mathbb {R}_+^N$. We have

$$\begin{aligned} A_{x, \sqrt{y}, z} (\zeta =0) = q. \end{aligned}$$

By subaveraging, we have

$$\begin{aligned} |F_j(q)|^p \le \frac{1}{2 \pi } \int _0^{2 \pi } |F_j (A_{x,\sqrt{y}, z} (e^{i t})) |^p \, dt. \end{aligned}$$

Now integrate the above inequality in (*z*, *x*) against $dx \, dv(z)$ (Haar measure for *M*) over any compact set $K \subset \mathbb {C}^n \times \mathbb {R}^m$; then take limits as $j \rightarrow \infty$; from Theorem 3.10 and the same integration argument in *z* and *x* that was just used, we obtain

30 $$\begin{aligned}&\int _{(z,x) \in K} |F(z, x+i \phi (z,z) +iy)|^p \, dx \, dv(z)\nonumber \\&\le \frac{1}{2 \pi } \int _{(z,x) \in K} \int _{t=0}^{2 \pi } |f(A_{x,\sqrt{y}, z} (e^{ i t})) |^p \, dt \, dx \, dv(z) \nonumber \\&\le \Vert f\Vert ^p_{L^p(M)}. \end{aligned}$$

Once we establish (19), the above inequality will prove (20). $\square$

### Proof of (19)

For the remainder of this section, we write $\phi (z)$ instead of $\phi (z,z)$ for simplicity. We will continue to use the analytic discs *Z*, *G* and $A_{x,\sqrt{y}, z}$ as defined in (21), (22), and (23), with $s=( \sqrt{y_1}, \dots , \sqrt{y_N}) \in \mathbb {R}_+^N$. We use a subaveraging and limiting argument applied to

$$\begin{aligned} F_j(A_{x,\sqrt{y}, z} (\zeta ))-f(z, x+i\phi (z)) \end{aligned}$$

similar to that used to establish (30) to conclude

31 $$\begin{aligned} \Big |\Big |F&(z, x+i\phi (z)+iy) - f(z, x+i\phi (z)) \Big |\Big |_{L^p(z,x)} \nonumber \\&\le \frac{1}{2 \pi } \int _{t=0}^{2 \pi }\Big |\Big | \left[ f(A_{x,\sqrt{y}, z}(e^{it}) )- f(z, x+i\phi (z)) \right] \Big |\Big |_{L^p(z,x)} \ dt \end{aligned}$$

where $\Vert \cdot \Vert _{L^p(z,x)}$ indicates the $L^p$-norm of a function defined on $\mathbb {C}^n\times \mathbb {R}^m$ in the (*z*, *x*) variables.

From (23) and (24), when $|\zeta |=1$:

$$\begin{aligned} A_{x,\sqrt{y}, z}(\zeta )&=\big (z+Z^0_{\sqrt{y}}(\zeta ), \ x+{{\,\textrm{Re}\,}}\{ G_{\sqrt{y}, z} (\zeta ) \}+i {{\,\textrm{Im}\,}}\{ G_{\sqrt{y}, z} (\zeta ) \} \big ) \\&= \big (z+Z^0_{\sqrt{y}}(\zeta ), \ x+{{\,\textrm{Re}\,}}\{ G_{\sqrt{y}, z} (\zeta ) \}+ i \phi (z+Z^0_{\sqrt{y}}(\zeta )) \big ). \end{aligned}$$

where the last equation uses the fact that the boundary of $A_{x, \sqrt{y},z}$ lies in *M*. This term is a translation of $(z, x+i\phi (z))$ in the *z* variable by $Z^0_{\sqrt{y}}(\zeta )$ and by ${{\,\textrm{Re}\,}}\{ G_{\sqrt{y}, z} (\zeta ) \}$ in the *x*-variable. From (21) and (22) with $s=\sqrt{y}$, we have

32 $$\begin{aligned} |Z^0_{\sqrt{y}}(\zeta )|&\le C|y|^{1/2} \end{aligned}$$

33 $$\begin{aligned} | {{\,\textrm{Re}\,}}\{ G_{\sqrt{y}, z} (\zeta ) \} |&\le C|y|^{1/2} |z| \quad \text {for small |{ y}|} \end{aligned}$$

where *C* is a uniform constant. Since translation is a continuous operator on functions in $L^p(z,x)$, we have the following: for a given $\varepsilon >0$, there is a $\delta >0$ so that if $|a|, |b| < \delta$, then

34 $$\begin{aligned} \Big |\Big | f(z+a, x+b +i \phi (z+a)) - f (z,x+i\phi (z)) \Big |\Big |_{L^p(z,x)} < \varepsilon /3. \end{aligned}$$

We also choose $R>0$ large enough so that

35 $$\begin{aligned} \Vert f(z,x+i\phi (z))\Vert _{L^p \{(z,x); \ |z| > R\}} < \varepsilon /3. \end{aligned}$$

Now from (31), we have

$$\begin{aligned} \Big |\Big |F&(z, x+i\phi (z)+iy) - f(z, x+i\phi (z)) \Big |\Big |_{L^p(z,x)} \\ \le&\frac{1}{2 \pi } \int _{t=0}^{2 \pi }\Big |\Big | \left[ f(A_{x,\sqrt{y}, z}(e^{it}) ) - f(z, x+i\phi (z)) \right] \Big |\Big |_{L^p \{(z,x) ; \ |z| \le R+1 \}} \ dt \\&+ \frac{1}{2 \pi } \int _{t=0}^{2 \pi }\Big |\Big | f(A_{x,\sqrt{y}, z}(e^{it}) ) \Big |\Big |_{L^p \{(z,x) ; \ |z|> R+1 \}} \ dt \\&+ \frac{1}{2 \pi } \int _{t=0}^{2 \pi } \Big |\Big | f(z, x+i\phi (z)) \Big |\Big |_{L^p \{(z,x) ; \ |z| > R+1 \}} \ dt\\ =&I+II+III \end{aligned}$$

By the choice of *R* (see (35), $III < \varepsilon /3$. For *II*, note that in view of (23) and the fact that $|Z_{z,\sqrt{y}}(\zeta )| \le C|y|^{1/2}$, if $|z|>R+1$, the *z*-component of $A_{x,\sqrt{y}, z}(\zeta )$ has modulus larger than *R* provided $C|y|^{1/2} \le 1$. Using (35), we obtain $II < \varepsilon /3$. Finally, in view of (33), we can further restrict $|y|>0$ small enough so that $C|y|^{1/2}|z| < \delta$ for all $|z| \le R+1$. The inequality in (34) then implies that $I < \varepsilon /3$. Therefore, we conclude that

$$\begin{aligned} \Big |\Big |F (z, x+i\phi (z)+iy) - f(z, x+i\phi (z)) \Big |\Big |_{L^p(z,x)} < \varepsilon \end{aligned}$$

and so $\lim _{y \rightarrow 0, y \in \Gamma _M} \Big |\Big |F (z, x+i\phi (z)+iy) - f(z, x+i\phi (z)) \Big |\Big |_{L^p(z,x)} =0$. Note that this limit is uniform in *y*. This completes the proof of Theorem 3.9.

**Step 4.** *Completion of the proof of Theorem *3.5. First, let us review steps 1–3. We started with a given $\tilde{f} \in H^p(M_1)$. In steps 1 and 2, we showed that $\tilde{f}$ has distribution boundary values on *M*, denoted by $f \in CR^p(M)$. In step 3, we showed that $CR^p(M)$ functions extend to $H^p(\overline{\Omega })$ functions. Let $F \in H^p(\overline{\Omega })$ be the extension of *f*. Since $\overline{\Omega }$ includes $M_1$ as part of its boundary, $\hat{f}:=F|_{M_1}$ is well-defined as an element of $H^p_{CR}(M_1)$. The uniqueness result in Step 2 (see Lemma 3.7) implies that $\hat{f} = \tilde{f}$ on $M_1$. Moreover, (10) and (11) follow from (19) and (20) since $\Gamma _M = \{(y_1, y_2 ) \in \mathbb {R}^2; \ y_1, y_2 \ge 0\}$ includes the vector $E_2 =(0,1)$.

The same reasoning proves the part of Theorem 3.5 where the starting point is $f \in CR^p(M)$. Such a function extends to an element $F \in H^p(\overline{\Omega })$. Then $\tilde{f}:=F|_{M_1}$ is well defined as an element of $H^p(M_1)$ which extends *f* and satisfies (10) and (11). The proof of Theorem 3.5 is now complete.

### Proof of Theorem 3.3

In Steps 1 and 2 above, an outline is given for how to generalize these steps for Theorem 3.5 (in which $N=m=2$) to the general context handled in Theorem 3.3. Moreover, Step 3 handles the general context. Thus, the proof of Theorem 3.3 is complete.

## The Bergman Kernel for $\Omega = {\text {int}}(M+i\Gamma _M)$

### Group Structure for a Quadric

We begin by reviewing the group structure on a quadric *M* as well as the explicit formulas for its Szegö kernel. We will first identify *M* with $\mathbb {C}^n \times \mathbb {R}^m$ via $(z,t) \mapsto (z, t+i \phi (z,z))$ for $(z,t) \in \mathbb {C}^n \times \mathbb {R}^m$. For $(z,t), (\zeta , u) \in \mathbb {C}^n \times \mathbb {R}^m$, define the group structure as follows:

36 $$\begin{aligned} (z,t)*(\zeta , u)=(z+\zeta , t+u -2 {{\,\textrm{Im}\,}}\phi (z, \zeta )). \end{aligned}$$

The lifting of this group operation to *M* is:

$$\begin{aligned} (z,w) *_M (\zeta , \eta ) = (z+\zeta , w+\eta + 2i \phi (z, \zeta )) \ \ \text {for }(z,w), (\zeta , \eta ) \in M. \end{aligned}$$

An easy computation shows that *M* is closed under $*_M$.

For $$(\zeta , u) \in \mathbb {C}^n \times \mathbb {R}^m$$,

$$\begin{aligned} (\zeta ,u)^{-1} = (-\zeta , -u). \end{aligned}$$

For later computations with convolution kernels, we will need to compute the following formula for the convolution product

37 $$\begin{aligned} (z,t)*(\zeta , u)^{-1} = (z-\zeta , t-u+2 {{\,\textrm{Im}\,}}\phi (z,\zeta )). \end{aligned}$$

The projection map $\pi : M \rightarrow \mathbb {C}^n \times \mathbb {R}^m$ given by $\pi (z, t+i \phi (z,z)) = (z,t)$ induces a CR structure on $\mathbb {C}^n \times \mathbb {R}^m$ given by the push forward of the CR vectorfields on *M*. (This is quite different from the trivial CR structure that $\mathbb {C}^n \times \mathbb {R}^m$ inherits as an imbedded subspace of $\mathbb {C}^n \times \mathbb {C}^m$). The map, $\pi$, is a CR isomorphism between the groups *M* and $\mathbb {C}^n \times \mathbb {R}^m$.

As defined earlier, the space of $L^2$ CR functions on *M* is denoted by $CR^2(M)$. Likewise, the space of $L^2$ CR functions on $\mathbb {C}^n \times \mathbb {R}^m$ is denoted by $CR^2(\mathbb {C}^n \times \mathbb {R}^m)$.

### The Szegö Kernel for *M* (and $\mathbb {C}^n \times \mathbb {R}^m$)

The Szegö operator for *M* is the orthogonal projection of $L^2(M)$ onto $CR^2(M)$. This operator is given by integration against a convolution kernel, on $M \times M$ denoted by $S(\alpha , \beta )$, for $\alpha , \beta \in M$. Likewise, the Szegö operator for $\mathbb {C}^n \times \mathbb {R}^m$ is the the orthogonal projection of $L^2(\mathbb {C}^n \times \mathbb {R}^m)$ onto $CR^2(\mathbb {C}^n \times \mathbb {R}^m)$. This operator is given by integration against a convolution kernel on $\{\mathbb {C}^n \times \mathbb {R}^m\} \times \{\mathbb {C}^n \times \mathbb {R}^m\}$ denoted by $S_0((z,t), \, (\zeta , u))$ for $(z,t), \, (\zeta , u) \in \mathbb {C}^n \times \mathbb {R}^m$. Since the measures on $\mathbb {C}^n \times \mathbb {R}^m$ and *M* are essentially the same (Haar measure, *dv*(*z*)*dv*(*t*)), and $\pi ^*(dv(z) dv(t))$, respectively) and since the graphing map $(z,t) \mapsto (z, t+i\phi (z,z))$ is a CR isomorphism, we have

38 $$\begin{aligned} S_0((z,t), \, (\zeta , u)) = S(\alpha , \beta ); \ \ \text {where } \alpha =(z, t+i \phi (z,z)), \ \beta = (\zeta , u+i \phi (\zeta , \zeta )) \in M. \end{aligned}$$

Our goal for this section is to write down a formula for the Szegö kernel for $M\times M$ and then extend this formula to $\Omega \times \Omega$ where $\Omega = \text {int} (M +i \Gamma _M)$. We will use this formula to construct the Bergman kernel for $\Omega \times \Omega$ for a certain class of cones, $\Gamma _M$.

In order to write down formulas for these Szegö kernels, we need to define the following quantities:

1. For $\nu \in S^{m-1}$ (the unit sphere in $\mathbb {R}^m$), define
  $$\begin{aligned} \phi _\nu (z, \zeta ) = \nu \cdot \phi (z, \zeta ). \end{aligned}$$
2. Since $\phi (z, \zeta )$ is a conjugate symmetric, bilinear form, there is a Hermitian symmetric $n \times n$ matrix $A^\phi _\nu$ such that
  $$\begin{aligned} \phi _\nu (z, \zeta ) = \zeta ^* A^\phi _\nu z, \ \ \text {for } z, \zeta \in \mathbb {C}^n. \end{aligned}$$
  The matrix function $\nu \mapsto A^\phi _\nu$ is a linear function of $\nu$.
3. Let $\mu _j^\nu$, $j=1, \dots , n$ be the (real) eigenvalues for $A^\phi _\nu$. Let $z^\nu = (z^\nu _1, \dots z^\nu _n) \in \mathbb {C}^n$ be the coordinates for *z* with respect to a frame which diagonalizes $A^\phi _\nu$. In particular
  $$\begin{aligned} \phi _\nu (z,z) = \sum _{j=1}^n \mu _j^\nu |z_j^\nu |^2. \end{aligned}$$
4. For the convex cone $\Gamma _M$ in $\mathbb {R}^m$, let $\Gamma _M^o$ be its dual cone, i.e.,
  $$\begin{aligned} \Gamma _M^o = \{ \nu \in S^{m-1}; \ \nu \cdot \gamma \ge 0, \text { for all }\gamma \in \Gamma _M \}. \end{aligned}$$

With these definitions, equation (25) with $q=0$ in [5] provides the following formula for the Szegö kernel $S_0$ for $\mathbb {C}^n \times \mathbb {R}^m$. For $(z,t) \in \mathbb {C}^n \times \mathbb {R}^m$,

39 $$\begin{aligned} S_0((z,t),0)&= C_{n,m} \int _{\nu \in \Gamma _M^o } \frac{\prod _{j=1}^n \mu _j^\nu }{\left( \sum _{j=1}^n \mu _j^\nu |z_j^\nu |^2 -i \nu \cdot t\right) ^{n+m}} \, d \nu \nonumber \\&\ \text {where }C_{n,m} = \frac{4^n(n+m-1)!}{(2 \pi )^{m+n}}\end{aligned}$$

40 $$\begin{aligned}&= C_{n,m} \int _{\nu \in \Gamma _M^o } \frac{ \det A^\phi _\nu }{(\phi _\nu (z,z) - i\nu \cdot t)^{n+m}} \, d \nu . \end{aligned}$$

Note that $\mu _j^\nu \ge 0$ for $1 \le j \le n$ since the domain of integration is restricted to $\{ \nu \in \Gamma _M^o\}$ and so $\phi _\nu (z,z) \ge 0$.

Now, the convolution kernel $S_0$ is obtained by inserting the formula for $(z,t)*(\zeta , u)^{-1}$ given in (37) in for (*z*, *t*) in (40). We obtain

41 $$\begin{aligned} S_0((z,t), \, (\zeta , u)) =C_{n,m} \int _{\nu \in \Gamma _M^o } \frac{ \det A^\phi _\nu \ d \nu }{(\phi _\nu (z- \zeta , z- \zeta ) -i \nu \cdot (t-u) -2i {{\,\textrm{Im}\,}}\phi _\nu (\zeta , u))^{n+m} }. \end{aligned}$$

Next, we wish to extend the integrand to the complex domain as an analytic function in $w=t+iy \in \mathbb {R}^m +i (\text {int} \, \Gamma _M)$ and a conjugate analytic function in $\eta =u+i v \in \mathbb {R}^m + i (\text {int} \, \Gamma _M)$. We obtain

42 $$\begin{aligned}&S_0((z,w), (\zeta , \eta ))\nonumber \\&\quad =C_{n,m} \int _{\nu \in \Gamma _M^o } \frac{\det A_\nu ^\phi \ d \sigma (\nu )}{\big (\phi _\nu (z\!-\!\zeta , z\!-\!\zeta ) \!+\!\nu \cdot (y+v) \!-\!i \nu \cdot (t-u) \!-\!2 i{{\,\textrm{Im}\,}}\phi _\nu (z, \zeta ) \big )^{n+m}} \end{aligned}$$

43 $$\begin{aligned}&\quad =C_{n,m} \int _{\nu \in \Gamma _M^o } \frac{\det A_\nu ^\phi \ d \sigma (\nu )}{(-2 \phi _\nu (\zeta , z) - i \nu \cdot [(w+i \phi (z,z)) - \overline{(\eta +i \phi (\zeta , \zeta ))}])^{n+m}}. \end{aligned}$$

Note that in (42), $\nu \cdot y>0$ and $\nu \cdot v>0$ for $y, v \in \text {int} \, \Gamma _M$ and so $S_0((z,w), (\zeta , \eta ))$ is well defined. From (43), we can identify $S(\alpha , \beta )$ extended to $\Omega \times \Omega$ (where $\Omega = \text {int} (M + i \Gamma _M)$) by using (38) with *t* and *u* replaced by *w* and $\eta$, respectively. We therefore define

44 $$\begin{aligned} S(\alpha , \beta ) := C_{n,m} \int _{\nu \in \Gamma _M^o } \frac{\det A_\nu ^\phi \ d \sigma (\nu )}{(-2 \phi _\nu (\zeta , z) -i \nu \cdot (w-\bar{\eta }))^{n+m}} \ \ \text {for } \alpha , \ \beta \in \Omega . \end{aligned}$$

Note that when $\alpha = (z,w)=(z, t+i(\phi (z,z)+y))$ and $\beta =(\zeta , \eta )=(\zeta , u+i(\phi (\zeta , \zeta )+v))$, we have $S(\alpha , \beta ) = S_0((z, t+iy), \, (\zeta , u+iv))$, which is consistent with (38). In particular, the extension of the Szegö kernel, $S(\alpha , \beta )$, to $\Omega \times \Omega$ can be described by either (42), (43), or (44).

As a consequence of (42), the following identities hold for $\alpha = (z,t+i \phi (z,z)+iy)$ and $\beta = (\zeta , u+i \phi (\zeta , \zeta ) +iv)$:

45 $$\begin{aligned} S(\alpha , \beta )&= S(\alpha -i(0,y), \beta +i(0,y)) \ \ \text {and} \end{aligned}$$

46 $$\begin{aligned} S(\alpha , \beta )&= S(\alpha +i(0, v), \beta -i(0,v)). \end{aligned}$$

### The Bergman Kernel for $\Omega$

To state and prove our theorem on the relationship between the Bergman kernel on $\Omega$ and the Szegö kernel on *M*, we must restrict the class of quadrics as specified in the following definition.

#### Definition 4.1

A quadric, $M \subset \mathbb {C}^n \times \mathbb {C}^m$ is *Bergman admissible* if there exists a basis of vectors, $\{V_1, \dots , V_m \}$, for $\mathbb {R}^m$ such that $\Gamma _M$ is the convex hull of $\{V_1, \dots , V_m \}$.

Note that this is a stronger condition than admissibility, as defined in Definition 2.1. By rescaling, we can assume the $V_j$ are unit vectors. To further simplify matters, we make a $\Box _b$-invariant linear change of coordinates in *w* and $\eta$ (see [4]) so that the vectors $V_1, \dots , V_m \in \mathbb {R}^m$ are the standard unit basis vectors $E_j$, with a 1 in the $j^{th}$ coordinate and zeros for the other coordinates. We call this the *model case*. Note that $\Gamma _M = \Gamma _M^o$ is the closed cone $\Gamma _Q$ in $\mathbb {R}^m$ given by

47 $$\begin{aligned} \Gamma _Q= \{(y_1, y_2, \dots , y_m) \in \mathbb {R}^m : \ y_j \ge 0, \ 1 \le j \le m \}. \end{aligned}$$

In this section and the next, we state and prove the following theorem generalizing the formula (2) of Nagel et al. [8]. After the proof, we use the standard change of variables formula to compute the Bergman kernel for the more general case of Bergman admissible quadrics (see Theorem 6.1).

#### Theorem 4.2

The Bergman kernel for the model case $\Omega = {\text {int}}(M+i\Gamma _Q)$ is given by

48 $$\begin{aligned} B(\alpha , \beta )= & {} (2 i)^m \frac{\partial ^m}{\partial \overline{ \eta }_1, \dots \partial \overline{\eta }_m } \Big \{ S(\alpha , (\zeta , \eta _1, \dots \, \eta _m)) \Big \} \nonumber \\{} & {} \text {for } \alpha =(z,w), \ \beta = (\zeta , \eta ) \in \Omega = {\text {int}}(M +i \Gamma _Q). \end{aligned}$$

Here, $S(\alpha , \beta )$ is given by formula (44).

#### Proof of Theorem 4.2

for general $m \ge 2$ is a repetition of the proof for $m=2$, which is the case we now consider. The strategy of the proof is to show that the function on the right-hand side of (48) has all three properties characterizing the Bergman kernel for $\Omega$. Thus our proof involves three steps:

Step 1. Convergence. Show that
  $$\begin{aligned} B(f)(\alpha ):= \int _{\beta \in \Omega } B(\alpha , \beta ) f(\beta ) \, d \beta \end{aligned}$$
  is well-defined for $\alpha \in \Omega$. Here, $d \beta$ is Lebesgue measure on $\Omega$.
Step 2. Reproducibility. Suppose $f \in A^2(\Omega )$, the space of square-integrable analytic functions on $\Omega$. Show that for all $\alpha \in \Omega$,
  $$\begin{aligned} f(\alpha ) = B(f)(\alpha ) = \int _{\beta \in \Omega } B(\alpha , \beta ) f(\beta ) \, d \beta . \end{aligned}$$
Step 3. Conjugate Symmetry. Show that for all $\alpha ,\beta \in \Omega$,
  $$\begin{aligned} B(\alpha , \beta )= \overline{ B(\beta , \alpha )}. \end{aligned}$$

Conjugate symmetry is easy to see from (44). As for convergence and reproducibility, we initially restrict the class of functions to $L^2(\Omega ) \cap H^2(\overline{\Omega })$, where $d \sigma (\beta ')$ is Haar measure on *M*. Because $L^2(\Omega ) \cap H^2(\overline{\Omega })$ is dense in $A^2(\Omega )$ (see Lemma 4.7 below), reproducibility will also hold for $A^2 (\Omega )$. $\square$

### Some Lemmas

Fix $V_0 = (1,1) \in {\text {int}} \Gamma _Q$. For $F \in L^2(\Omega ) \cap H^2(\overline{\Omega })$, define

$$\begin{aligned} F_{\varepsilon }(\alpha ):=F(\alpha +i \varepsilon V_0). \end{aligned}$$

Note that $F_{\varepsilon }$ is analytic on $\Omega _\varepsilon :=\Omega - i \varepsilon V_0$, a set that contains $\overline{ \Omega }$.

#### Lemma 4.3

Fix $V \in \Gamma _Q$ and let $F \in L^2(\Omega ) \cap H^2(\overline{\Omega })$. For each fixed $r \in \mathbb {R}^+$, the function

$$\begin{aligned} \alpha \in \Omega \mapsto F_\varepsilon (\alpha + i r V) \end{aligned}$$

belongs to $$L^2(\Omega ) $$ and the $$L^2 $$ norms converge to zero as $$r \rightarrow \infty $$.

#### Proof

This result is elementary and only uses the fact that *F* belongs to $L^2 (\Omega )$. $\square$

#### Lemma 4.4

Let $F \in L^2(\Omega ) \cap H^2(\overline{\Omega })$. For almost every $\varepsilon >0$, the functions

$$\begin{aligned} (\alpha ', r) \in M \times \mathbb {R}^+ \mapsto F_\varepsilon (\alpha ' +i rE_1) \ \ \text {and} \ \ (\alpha ', r) \in M \times \mathbb {R}^+ \mapsto F_\varepsilon (\alpha ' +i rE_2) \end{aligned}$$

are $$L^2 $$ functions on $$M \times \mathbb {R}^+$$.

#### Proof

Recall that

$$\begin{aligned} \overline{\Omega } = \{\alpha ' + i s E_1 + ir E_2: \alpha ' \in M, s, r \ge 0\}, \end{aligned}$$

so $$\overline{\Omega }$$ contains

$$\begin{aligned} \{\alpha '+i\varepsilon V_0 +i rE_2: \varepsilon , r \ge 0 \}. \end{aligned}$$

Thus if $F \in L^2(\Omega )$, the following integrals are finite:

$$\begin{aligned} \int _\Omega |F(\alpha )|^2 \,d\alpha&\ge \int _0^\infty \left[ \int _0^\infty \int _M |F(\alpha ' + i \varepsilon V_0 +i r E_2)|^2 \,d\sigma (\alpha ')\,d r \right] \,d \varepsilon \\&= \int _0^\infty \left[ \int _0^\infty \int _M |F_\varepsilon (\alpha ' +i r E_2)|^2 \,d\sigma (\alpha ')\,d r \right] \,d \varepsilon . \end{aligned}$$

Thus for almost every $\varepsilon$, the integral in square brackets is finite, proving the claim. $\square$

#### Lemma 4.5

Let *D* be any differential operator of arbitrary order involving $\frac{\partial }{\partial z_j}$ and $\frac{\partial }{\partial w_k}$. Let $F \in L^2(\Omega ) \cap H^2(\overline{\Omega })$. Then for every $\varepsilon >0$, $DF_\varepsilon$ also belongs to $L^2(\Omega ) \cap H^2(\overline{\Omega })$.

#### Proof

By induction, we can assume that the differential operator is of order one, which reduces the proof to one complex variable. Thus assume $\Omega$ is the upper half plane in $\mathbb {C}$ and suppose $F \in L^2(\Omega ) \cap H^2(\overline{\Omega })$. Fix $\varepsilon >0$. Then $F_\varepsilon (z) = F(z+i\varepsilon )$ and $D = \frac{\partial }{\partial z}$. Observe that $F_\varepsilon$ is analytic in $\{ {{\,\textrm{Im}\,}}z>-\varepsilon \}$. We must show $D F_\varepsilon \in L^2(\Omega ) \cap H^2(\overline{\Omega })$.

We first show that $DF_\varepsilon$ belongs to $L^2(\Omega )$. Suppose $z = x+iy$ with $y>0$. By the Cauchy Integral Formula, for $\rho < y+\varepsilon$,

$$\begin{aligned} |DF_\varepsilon (z) | \le \frac{1}{2\pi \rho } \int _0^{2 \pi } | F_\varepsilon (z+\rho e^{i\theta }) | \,d\theta . \end{aligned}$$

Multiplying by $\rho ^2$ and integrating in $\rho$ from 0 to *r* gives

$$\begin{aligned} |DF_\varepsilon (z)| \cdot \frac{r^3}{3}&\le \frac{1}{2 \pi } \int _0^r \int _0^{2 \pi } |F_\varepsilon (z+\rho e^{i \theta })| \rho \,d \theta d\rho \\&=\frac{1}{2\pi } \int _{D(z,r)} |F_\varepsilon ( z' ) | \,dA\\&\le C r \left( \int _{D(z,r)} |F_\varepsilon (z')|^2 dA \right) ^\frac{1}{2}, \end{aligned}$$

where we have used Cauchy-Schwarz at the last step. Thus

$$\begin{aligned} |DF_\varepsilon (z)|^2&\le \frac{C}{r^4} \int _{D(z,r)} |F_\varepsilon (z')|^2 \,dA\\&\le \frac{C}{r^4} \int _{|x'-x| \le r} \int _{|y'-y| \le r} |F_\varepsilon (x'+iy')|^2 \,dy' \,dx' \\&= \frac{C}{r^4} \int _{|t| \le r} \int _{|s| \le r } |F_\varepsilon (x+t+i(y+s))|^2 \, ds \, dt. \end{aligned}$$

Take $r = \varepsilon$ and integrate in *x* and *y* to get a bound on $\Vert DF_\varepsilon \Vert _{L^2(\Omega )}$.

$$\begin{aligned} \Vert DF_\varepsilon \Vert ^2_{L^2(\Omega )}&\le \frac{C}{\varepsilon ^4} \int _{|t| \le \varepsilon } \int _{|s| \le \varepsilon } \left[ \int _0^\infty \int _{-\infty }^\infty |F(x+t+i(y+s+\varepsilon ))|^2 \,dx\,dy \right] \,ds\,dt \\&\le \frac{C}{\varepsilon ^4} \int _{|t| \le \varepsilon } \int _{|s|\le \varepsilon } \left[ \int _0^\infty \int _{-\infty }^\infty |F(x'+iy')|^2 \,dx' \,dy' \right] \,ds\,dt\\&= \frac{C}{\varepsilon ^2}\Vert F\Vert ^2_{L^2(\Omega )}. \end{aligned}$$

Next we show that $DF_\varepsilon$ belongs to $H^2(\overline{\Omega } )$. Suppose $y={{\,\textrm{Im}\,}}z>0$. As above,

$$\begin{aligned} |DF_\varepsilon (x+iy)|^2 \le \frac{C}{\varepsilon ^4} \int _{|x'-x| \le \varepsilon } \int _{|y'-y| \le \varepsilon } |F_\varepsilon (x'+i y')|^2 \, d x' \, dy'. \end{aligned}$$

Let $t=x'-x$. Then integrate in *x* to obtain

$$\begin{aligned} \int _{-\infty }^\infty |DF_\varepsilon (x+iy)|^2 \, dx&\le \frac{C}{\varepsilon ^4} \int _{|t| \le \varepsilon } \int _{-\infty }^\infty \int _{|y-y'| \le \varepsilon } |F_\varepsilon (x+t+iy')|^2 \,dy' \,dx \,dt \\&=\frac{C}{\varepsilon ^4} \int _{|t| \le \varepsilon } \int _{-\infty }^\infty \int _{|y-y'| \le \varepsilon } |F_\varepsilon (x'+iy')|^2 \,dy' \,dx' \,dt\\&= \frac{C}{\varepsilon ^3} \int _{-\infty }^\infty \int _{|y-y'| \le \varepsilon } |F_\varepsilon (x'+iy')|^2 \,dy' \,dx'\\&\le \frac{C}{\varepsilon ^2} \sup _{|y'-y|\le \varepsilon } \int _{-\infty }^\infty |F_\varepsilon (x'+iy')|^2 \,dx'. \end{aligned}$$

Because $|y'-y| \le \varepsilon$, $y'+\varepsilon \ge y >0$ and so the last expression is bounded by $\frac{C}{\varepsilon ^2} \Vert F\Vert _{H^2(\overline{\Omega })}$. Taking the supremum over all $y>0$ shows $DF_\varepsilon$ belongs to $H^2(\overline{\Omega })$. $\square$

#### Lemma 4.6

Let $F \in L^2(\Omega ) \cap H^2(\overline{\Omega })$. Then for $V \in \Gamma _Q$,

49 $$\begin{aligned} \int _{\beta ' \in M} S(\alpha , \beta ') F_\varepsilon (\beta '+iV)\, d \beta '&=F_\varepsilon (\alpha +iV) \end{aligned}$$

50 $$\begin{aligned} \int _{\beta ' \in M} S(\alpha , \beta '+iV) F_\varepsilon (\beta ')\, d \beta '&=F_\varepsilon (\alpha +iV) \end{aligned}$$

In addition, the map

$$\begin{aligned} G \in L^2(M) \mapsto \int _{\beta ' \in M} S(\alpha , \beta ') G(\beta ') \, d \beta ' \end{aligned}$$

is a continuous map from $$L^2(M) $$ to $$H^2 (\overline{\Omega })$$.

#### Proof

Formula (49) follows from the reproducibility of *S* and the fact that $F_\varepsilon (\alpha +iV)$ is a CR function (in $\alpha$) on *M*. From (42), $S(\alpha , \beta '+iV) = S(\alpha +iV, \beta ')$ from which (50) follows. The last statement follows from the fact that the operator *S* is a continuous $L^2$ projection onto CR$^2(M)$ which in turn is isomorphic to $H^2(\overline{\Omega })$ in view of the $H^2$ result in Theorem 3.9. $\square$

#### Lemma 4.7

$L^2(\Omega ) \cap H^2(\overline{\Omega })$ is dense in $A^2(\Omega )$.

#### Proof

Let $F \in A^2(\Omega )$. As above, set

$$\begin{aligned} F_\varepsilon (\alpha ) = F \left( z,t+i(y+\phi (z,z) + \varepsilon V_0)\right) . \end{aligned}$$

Because *F* is analytic on $\Omega$, so is $F_\varepsilon$. Moreover, $F_\varepsilon |_M$ is a CR function because $F_\varepsilon$ is analytic in an open set containing *M*. Fix $y\in \Gamma _Q$ and set $r=(r_1,r_2)= \frac{1}{2}\varepsilon V_0+y$. Let $P(w;r_1,r_2)$ be the closed (Euclidean) polydisc $\overline{D(w_1,r_1)}\times \overline{D(w_2,r_2)}$. The factor of 1/2 ensures that the polydisc lies within the interior of the set where $F_\varepsilon$ is analytic in the computation below. By the Mean Value Property for analytic functions, for $w = t+i(\phi (z,z)+r)$ fixed and $\eta = u+iv$,

$$\begin{aligned} F_\varepsilon (\alpha )&= F_\varepsilon \left( z, t+i(\phi (z,z)+r) \right) \\&= \frac{1}{\pi ^2 r_1^2 r_2^2} \int _{\eta \in P(0;r_1,r_2)} F_\varepsilon \left( z, t+u+i(r + v + \phi (z,z))\right) \, du\, dv. \end{aligned}$$

Consequently, by Cauchy-Schwarz,

$$\begin{aligned} |F_\varepsilon (\alpha )| \le \frac{1}{\pi r_1r_2}\left( \int _{\eta \in P(0;r_1,r_2)} \left| F_\varepsilon \left( z, t+u+i(r + v + \phi (z,z))\right) \right| ^2\, du\, dv\right) ^{\frac{1}{2}} \end{aligned}$$

so that

$$\begin{aligned} |F_\varepsilon (\alpha )|^2 \le \frac{1}{\pi ^2 r_1^2r_2^2}\int _{\eta \in P(0;r_1,r_2)} \left| F_\varepsilon \left( z, t+u+i(r + v + \phi (z,z))\right) \right| ^2\, du\, dv \end{aligned}$$

and therefore

$$\begin{aligned}&\int _{\mathbb {C}^n\times \mathbb {R}^2} \left| F_\varepsilon \left( z,t+ i r + i\phi (z,z)\right) \right| ^2\, dz\, dt\\&\le \frac{1}{\pi ^2 r_1^2r_2^2} \int _{(z,t)\in \mathbb {C}^n\times \mathbb {R}^2}\int _{\eta \in P(0;r_1,r_2)} \left| F_\varepsilon \left( z, t+u+i(r + v + \phi (z,z))\right) \right| ^2\, du\, dv\, dz\, dt \\&\le \frac{1}{\pi ^2 r_1^2r_2^2} \int _{\genfrac{}{}{0.0pt}2{|u_1|\le r_1}{|u_2|\le r_2}}\int _{(z,t+iv+i\phi (z,z))\in \Omega } \left| F_\varepsilon \left( z, t+u+i(r + v + \phi (z,z))\right) \right| ^2\, dv\, dz\, dt\, du \\&\le \frac{C \Vert F_\varepsilon \Vert _{L^2(\Omega )}^2}{r_1r_2}. \end{aligned}$$

Because $r=\frac{1}{2}\varepsilon V_0+y$, this calculation shows that

$$\begin{aligned} \int _{\mathbb {C}^n\times \mathbb {R}^2} \left| F_{3\varepsilon /2}\left( z,t+ i y + i\phi (z,z)\right) \right| ^2\, dz\, dt \le \frac{C \left| F_\varepsilon \right| _{L^2(\Omega )}^2}{\left( \frac{1}{2}\varepsilon +y_1\right) \left( \frac{1}{2}\varepsilon +y_2\right) }. \end{aligned}$$

Thus $F_\varepsilon \in H^2(\overline{\Omega })$, $\Vert F_\varepsilon \Vert _{H^2(\overline{\Omega })} \le \varepsilon ^{-1}\Vert F\Vert _{L^2(\Omega )}$ (a useful estimate for *y* small), and

$$\begin{aligned} \int _{\mathbb {C}^n\times \mathbb {R}^2} \left| F_\varepsilon \left( z,t+ ir + i\phi (z,z)\right) \right| ^2\, dz\, dt \le \frac{\Vert F\Vert ^2_{L^2(\Omega )}}{y_1y_2}, \end{aligned}$$

(a useful estimate for *y* large). The lemma now follows from the fact that $F_\varepsilon \rightarrow F$ in $L^2(\Omega )$, as $\varepsilon \rightarrow 0$. $\square$

## The Proof of Theorem 4.2

As already mentioned, the kernel $B(\alpha , \beta )$, as defined in (48), is conjugate symmetric. It remains to show that *B* reproduces $F \in A^2(\Omega )$. In view of Lemma 4.7, it suffices to show reproducibility for $F_\varepsilon$ when $F \in L^2(\Omega ) \cap H^2(\overline{\Omega })$.

For $F \in L^2(\Omega ) \cap H^2(\overline{\Omega })$, $F_\varepsilon$ is an $L^2$ CR function on *M* and so

$$\begin{aligned} \int _{\beta ' \in M} S(\alpha , \beta ') F_\varepsilon (\beta ' ) \, d \beta ' = F_\varepsilon (\alpha ) \end{aligned}$$

for $\alpha \in \Omega$. We first show the following lemma:

### Lemma 5.1

For any $$F \in L^2(\Omega ) \cap H^2(\overline{\Omega })$$,

51 $$\begin{aligned} 2i \int _{\beta \in M_1} \frac{\partial S(\alpha , \beta )}{\partial \bar{\eta }_2} F_\varepsilon (\beta ) \, d \beta = F_\varepsilon (\alpha ), \ \ \text {for}\; \alpha \in \Omega . \end{aligned}$$

Once this lemma is established, we then repeat this step, only one dimension higher, and show

52 $$\begin{aligned} -4 \int _{\beta \in \Omega } \frac{\partial ^2 S(\alpha , \beta )}{\partial \bar{\eta }_1\partial \bar{\eta }_2} F_\varepsilon (\beta ) \, d \beta = F_\varepsilon (\alpha ) \end{aligned}$$

for $$\alpha \in \Omega $$.

### Remark 5.2

Equation (51) actually holds for any $F_1 \in CR^2(M_1)$. This can be shown by proving the analogue of Lemma 4.7 that says that $H^2_{CR}(M_1) \cap L^2(M_1)$ is dense in $CR^2(M_1)$. This fact together with conjugate symmetry of the kernel $2i\frac{\partial S(\alpha , \beta )}{\partial \bar{\eta }_2}$ shows that $2i\frac{\partial S(\alpha , \beta )}{\partial \bar{\eta }_2}$ is the kernel representing the projection operator onto $CR^2(M_1)$.

### Proof of Lemma 5.1

The proof of (51) involves integration by parts. We begin by establishing some notation. Recall that $M_1$ is parameterized by

$$\begin{aligned}{} & {} (\zeta , u, v_2) \in \mathbb {C}^n \times \mathbb {R}^2 \times \mathbb {R}^+ \mapsto \beta :=\beta ' +(0, 0, iv_2) = (\zeta , u+i\phi (\zeta , \zeta ) \\{} & {} +i(0,v_2)) \in M+(0, 0, i\mathbb {R}^+). \end{aligned}$$

We also write $\eta = u+iv \in \mathbb {C}^2$. By a slight abuse of notation, we write $\beta =\beta '+iv_2$.

Next, fix $R, R'>0$ and define

$$\begin{aligned} M_1(R, R')&:= \{ \beta =\beta '+iv_2 \in M_1 : 0 \le v_2 \le R \ \text {and} \ |u_2| \le R' \}\\ M(R')&:= \{ \beta ' \in M : |u_2| \le R' \}. \end{aligned}$$

We now prove (51). We integrate by parts in $v_2$. Since *S* is conjugate analytic in $\eta _2=u_2+iv_2$,

$$\begin{aligned} \frac{\partial S(\alpha , \beta )}{\partial \bar{\eta }_2} = i\frac{\partial S(\alpha , \beta )}{\partial v_2}, \end{aligned}$$

and so we obtain

$$\begin{aligned}&\int _{\beta \in M_1 (R, R')} \frac{\partial S(\alpha , \beta )}{\partial \bar{\eta }_2} F_\varepsilon (\beta ) \, d \beta \\&= i \int _{v_2=0}^R \int _{\beta ' \in M(R')} \frac{\partial S(\alpha , \beta '+iv_2)}{\partial v_2} F_\varepsilon (\beta '+iv_2) \,d\beta ' dv_2\\&=-i \int _{v_2=0}^R \int _{\beta ' \in M(R')} S(\alpha , \beta '+iv_2) \frac{\partial F_\varepsilon (\beta '+iv_2 ) }{\partial v_2} \, d \beta ' \, dv_2 \\&+i \int _{\beta ' \in M(R')} S(\alpha , \beta '+iR) F_\varepsilon (\beta ' +iR) d \beta '\\&- i \int _{\beta ' \in M(R')} S(\alpha , \beta ') F_\varepsilon (\beta ') \, d \beta ' = A+B+C. \end{aligned}$$

Lemma 4.6 implies that as $R' \rightarrow \infty$, *B* converges in $H^2(\overline{\Omega })$ to $iF_\varepsilon (\alpha +2iR)$ and *C* converges to $-i F_\varepsilon (\alpha )$. Furthermore, by Lemma 4.5, $\frac{\partial F_\varepsilon }{\partial v_2}$ belongs to $L^2(\Omega ) \cap H^2(\overline{\Omega })$, and so the inner $\beta '$ integral in *A* converges in $H^2(\overline{\Omega })$ to $\frac{\partial F_\varepsilon (\alpha +2iv_2)}{\partial v_2}$. Thus, we can let $R' \rightarrow \infty$ and the above equation becomes

53 $$\begin{aligned} \int _{\beta \in M_1 (R, \infty )} \frac{\partial S(\alpha , \beta )}{\partial \bar{\eta }_2} F_\varepsilon (\beta ) \, d \beta =&-i \int _0^R \int _{\beta ' \in M} S(\alpha , \beta '+iv_2) \frac{\partial F_\varepsilon (\beta '+iv_2 ) }{\partial v_2} \, d \beta ' \, dv_2 \nonumber \\&+ i F_\varepsilon (\alpha +2iR) -i F_\varepsilon (\alpha ). \end{aligned}$$

We now use the Cauchy-Riemann equations to convert $-i\frac{\partial F_\varepsilon }{\partial v_2}$ to $\frac{\partial F_\varepsilon }{\partial u_2}$. We then integrate by parts in $u_2$ in the first term on the right. We claim

54 $$\begin{aligned} \int _{\beta \in M_1 (R, \infty )} \frac{\partial S(\alpha , \beta )}{\partial \bar{\eta }_2} F_\varepsilon (\beta ) \, d \beta =&\int _0^R \int _{\beta ' \in M} S(\alpha , \beta '+iv_2) \frac{\partial F_\varepsilon (\beta '+iv_2 ) }{\partial u_2} \, d \beta ' \, dv_2 \nonumber \\&+ i F_\varepsilon (\alpha +2iR) -i F_\varepsilon (\alpha ) \end{aligned}$$

55 $$\begin{aligned} =&- \int _0^R \int _{\beta ' \in M} \frac{\partial S(\alpha , \beta '+iv_2)}{\partial u_2} F_\varepsilon (\beta '+iv_2 ) \, d \beta ' \, dv_2 \nonumber \\&+ i F_\varepsilon (\alpha +2iR) -i F_\varepsilon (\alpha ) . \end{aligned}$$

We must justify that there are no boundary terms. To this end, let $R'>>0$ and let $\psi _{R'}$ be a smooth real-valued function of $u_2$ satisfying

$$\begin{aligned} \psi _{R'} (u_2) = {\left\{ \begin{array}{ll} 1 &{} \text { if } |u_2| \le R' \\ 0 &{} \text { if } |u_2| \ge R'+1. \end{array}\right. } \end{aligned}$$

Then

56 $$\begin{aligned} \int _0^R \int _{\beta ' \in M}&S(\alpha , \beta '+iv_2) \frac{\partial F_\varepsilon (\beta '+iv_2 )}{\partial u_2} \psi _{R'} (u_2) \, d \beta ' \, dv_2 \end{aligned}$$

57 $$\begin{aligned} =&- \int _0^R \int _{\beta ' \in M} \frac{\partial S(\alpha , \beta '+iv_2)}{\partial u_2} F_\varepsilon (\beta '+iv_2 ) \psi _{R'} (u_2) \, d \beta ' \, dv_2 \end{aligned}$$

58 $$\begin{aligned}&- \int _0^R \int _{\beta ' \in M, \ R' \le |u_2| \le R'+1} S(\alpha , \beta '+iv_2) F_\varepsilon (\beta '+iv_2 ) \frac{ \partial \psi _{R'} (u_2)}{\partial u_2} \, d \beta ' \, dv_2. \end{aligned}$$

We claim that by the Dominated Convergence Theorem, for each fixed *R*, the $v_2$-integral in (56) converges in $L^2(\alpha ' \in M)$, as $R' \rightarrow \infty$, to the corresponding integral on the right side of (54). Indeed, fix any $R>0$ and any $\varepsilon >0$ satisfying Lemma 4.4. For each $R' >0$, $v_2 \ge 0$, and $\alpha ' \in M$, define

$$\begin{aligned} Sz (\alpha ',R', v_2)= \int _{\beta ' \in M} S(\alpha ', \beta ' +iv_2 ) \frac{\partial F_\varepsilon (\beta '+iv_2)}{\partial u_2} \psi _{R'} (u_2) \, d \beta '. \end{aligned}$$

Note that $Sz(\alpha ',R', v_2)$ represents the value at $\alpha '+iv_2$ of the $H^2$-extension of the Szegö projection of the function

$$\begin{aligned} \beta ' \in M \mapsto \frac{\partial F_\varepsilon (\beta '+iv_2)}{\partial u_2} \psi _{R'} (u_2). \end{aligned}$$

In particular, the $L^2(M)$-norm of *Sz* as an operator is at most 1 (because projection and $H^2$ extension operators both have norm $\le 1$). This implies

$$\begin{aligned} \big \Vert Sz( \alpha ', R', v_2)\big \Vert _{L^2(\alpha ' \in M)}&\le \Big \Vert \frac{\partial F_\varepsilon (\beta '+iv_2)}{\partial u_2} \psi _{R'} (u_2)\Big \Vert _{L^2(\beta ' \in M)} \\&\le \Big \Vert \frac{\partial F_\varepsilon (\beta '+iv_2)}{\partial u_2}\Big \Vert _{L^2(\beta ' \in M)} \end{aligned}$$

for all $$R'>0$$ and all $$0 \le v_2 \le R$$. Lemma 4.5 implies that $$\frac{\partial F_\varepsilon }{\partial u_2}$$ belongs to $$H^2(\overline{\Omega })$$. Since $$\varepsilon $$ satisfies the conclusion of Lemma 4.4, the right side is an $$L^2 $$ function in $$v_2 \ge 0$$.

The paragraph immediately following (58) shows that for each $0 \le v_2 \le R$,

59 $$\begin{aligned} \ \ \ \lim _{R' \rightarrow \infty } Sz (\alpha ', R', v_2) =\int _{\beta ' \in M} S(\alpha ', \beta ' +iv_2 ) \frac{\partial F_\varepsilon (\beta '+iv_2)}{\partial u_2} \, d \beta '. \end{aligned}$$

Thus by the Dominated Convergence Theorem,

$$\begin{aligned} \lim _{R' \rightarrow \infty } \int _{v_2=0}^R Sz (\alpha ', R', v_2) \, dv_2 = \int _{v_2=0}^R \int _{\beta ' \in M} S(\alpha ', \beta ' +iv_2 ) \frac{\partial F_\varepsilon (\beta '+iv_2)}{\partial u_2} \, d \beta ' \, dv_2, \end{aligned}$$

where the convergence is in $L^2(\alpha ' \in M)$. A similar argument shows that (58) converges in $L^2(\alpha ' \in M)$ to zero as $R' \rightarrow \infty$. This forces (57) to converge as $R' \rightarrow \infty$ to the term on the right side of (54). Thus, the integration by parts calculation to obtain (55) from (54) is justified.

Now observe that $\frac{\partial S(\alpha , \beta '+iv_2)}{\partial u_2}= \frac{\partial S(\alpha , \beta '+iv_2)}{\partial \bar{\eta }_2}$. Thus the first term on the right side of (55) can be absorbed into the left side of this equation to obtain

$$\begin{aligned} \int _{\beta \in M_1 (R, \infty )} \frac{\partial S(\alpha , \beta )}{\partial \bar{\eta }_2} F_\varepsilon (\beta ) \, d \beta =\frac{ i}{2} F_\varepsilon (\alpha +2iR) -\frac{i }{2}F_\varepsilon (\alpha ). \end{aligned}$$

Now let $R \rightarrow \infty$ and note that $F_\varepsilon (\alpha +2iR) \rightarrow 0$ in $L^2(\Omega )$ (Lemma 4.3). Therefore

$$\begin{aligned} \int _{\beta \in M_1 } \frac{\partial S(\alpha , \beta )}{\partial \bar{\eta }_2} F_\varepsilon (\beta ) \, d \beta = -\frac{i }{2}F_\varepsilon (\alpha ), \ \ \alpha \in \Omega \end{aligned}$$

and (51) follows. The proof of Lemma 5.1 is complete. $\square$

Observe that (51) establishes that $\frac{\partial S(\alpha , \beta )}{\partial \bar{\eta }_2}$ is a reproducing kernel for $F_\varepsilon \in H^2 (\overline{\Omega }) \cap L^2(\Omega )$ over $M_1$. In addition, this kernel is conjugate symmetric and thus defines an orthogonal projection operator. So repeating the above argument with $S(\alpha , \beta )$ replaced by $\frac{\partial S(\alpha , \beta )}{\partial \bar{\eta }_2}$, *M* replaced by $M_1$, and $M_1$ replaced by $\overline{\Omega } = M_1+i\{v_1 \ge 0 \}$ establishes (52) for $F_\varepsilon \in H^2(\overline{\Omega })$. Lemma 4.7 then implies that (52) holds for $F \in A^2(\Omega )$. This concludes the proof of Theorem 4.2 in the case $m=2$. The proof for $m>2$ involves further iterations of the same argument used to establish the case for $m=2$.

## The Bergman Kernel for $\Omega = {\text {int}}(M+i\Gamma _M)$: The General Case

In this section, we use the formula for the Bergman kernel in the model case, (48), together with the standard change of variables formula for the Bergman kernel under a biholomorphic map that takes the model case to the general case. The main theorem of this section is as follows:

### Theorem 6.1

Assume *M* is a Bergman admissible quadric with $\Gamma _M$ equal to the convex hull of a set of basis vectors $\{V_1, \dots , V_m\}$ for $\mathbb {R}^m$. Let $\phi$ be the Hermitian symmetric quadratic form for *M* and suppose $\Omega = {\text {int}}(M+i\Gamma _M)$. Let *V* be the $m \times m$ matrix whose $j^{th}$ column is the vector $V_j$, $1 \le j \le m$, and let $A^\phi _\nu$ be the complex Hessian matrix for $\phi _\nu = \phi \cdot \nu$. The Bergman kernel for $\Omega$ is

60 $$\begin{aligned} B_{\Omega }(\alpha , \beta )&= \frac{ \tilde{C}_{n,m}}{ |\det V|} \int _{\nu \in \Gamma ^o_M} \frac{(\det A^\phi _\nu ) \, \prod _{j=1}^m ( V_j \cdot \nu ) \, d \sigma (\nu )}{ \left[ 2 \phi _\nu (z, \zeta )+i \nu \cdot (w-\bar{\eta }) \right] ^{n+2m} } \nonumber \\ \tilde{C}_{n,m}&=\frac{2^{n}(-1)^m(n+2m-1)!}{ \pi ^{m+n}}; \ \ d \sigma (\nu ) = \text {surface measure for } S^{m-1} \end{aligned}$$

where $\Gamma _{M}^o = \{ \nu \in S^{m-1}; \ \nu \cdot \gamma \ge 0,$ for all $\gamma \in \Gamma _M \}$ is the dual cone for $\Gamma _M$.

### Remark 6.2

Note that the formula on the right side of (60) is independent of the magnitude of the vectors $V_j$ in view of the $\det V$ appearing in the denominator.

### Proof

Let $\Omega '$ be the model quadric domain in $\mathbb {C}^n \times \mathbb {C}^m$ with quadric edge $M'$ as defined in Sect. 4.3. The quadratic form associated with $M'$ will be denoted $\phi '$. Here, $\Gamma _{M'}$ is the cone $\Gamma _Q$ defined in (47). In addition, $A^{\phi '}_{\nu '}$ is the Hermitian symmetric matrix satisfying $\nu ' \cdot \phi '(z',\zeta ') = \zeta '^* A^{\phi '}_{\nu '} z'$ for $z', \zeta \in \mathbb {C}^n$. Then, formulas (48) and differentiation of (44) provide the following expression for the Bergman kernel in $\Omega '$.

61 $$\begin{aligned} B_{\Omega '}(\alpha ' , \beta ') = \frac{2^n (n+2m-1)!}{\pi ^{m+n}} \int _{ \nu ' \in \Gamma ^o_{M'}}\frac{\det (A^{\phi '}_{\nu '}) (-i \nu '_1) \dots (-i \nu '_m) \, d \sigma (\nu ')}{\left[ 2 \phi '_{\nu '}(z', \zeta ')+i \nu ' \cdot (w'-\bar{\eta }') \right] ^{n+2m}}. \end{aligned}$$

By abuse of notation, let $V: \mathbb {C}^m \rightarrow \mathbb {C}^m$ be the linear map associated with the $m \times m$ matrix *V*. Note that $\widetilde{V}: \mathbb {C}^n \times \mathbb {C}^m \rightarrow \mathbb {C}^n \times \mathbb {C}^m$ defined by $\widetilde{V}(z,w):= (z, V(w))$ is a biholomorphic map between the model $\Omega '=M'+i\Gamma _Q$ and $\Omega = M+ i \Gamma _M$.

We now recall the well-known change of variables formula for the Bergman kernel.

$$\square $$

### Lemma 6.3

Let $F:\Omega ' \rightarrow \Omega$ be a biholomorphism between two open sets in $\mathbb {C}^N$. Let $\alpha = F(\alpha ')$ and $\beta = F(\beta ')$. Then

$$\begin{aligned} B_{\Omega '} (\alpha ', \beta ') = B_\Omega (\alpha ,\beta ) \det F'(\alpha ') \overline{\det F'(\beta ')}. \end{aligned}$$

We apply this lemma to $\Omega ' = {\text {int}}(M'+i \Gamma _Q)$, $\Omega ={\text {int}}(M+i\Gamma _M)$, and the map $F= \widetilde{V}$, i.e.,

$$\begin{aligned} F(\alpha ')&=\tilde{V}(z', w') = (z', V w')=(z, w) = \alpha \ \ \text { and} \\ F(\beta ')&=\tilde{V} (\zeta ', \eta ') = (\zeta ', V \eta ') = (\zeta , \eta ) = \beta . \end{aligned}$$

Note that $\det F'(\alpha ')$ and $\det F'(\beta ')$ equal the real constant, $\det V$. Therefore

62 $$\begin{aligned} B_\Omega (\alpha ,\beta ) =\frac{1}{(\det V)^2} B_{\Omega '} (\alpha ', \beta '). \end{aligned}$$

We now compute $B_{\Omega '} (\alpha ', \beta ')$ using (61). We will need the following:

$$\begin{aligned} \alpha '&= (z',w') =F^{-1}(\alpha )= (z, V^{-1}w) \\ \beta '&=(\zeta ',\eta ') = F^{-1} (\beta ) = (\zeta , V^{-1} \eta ). \end{aligned}$$

Also define

63 $$\begin{aligned} \nu = \frac{(V^{-1})^t ( \nu ')}{|(V^{-1})^t ( \nu ')|} \in S^{m-1}. \end{aligned}$$

Note that

64 $$\begin{aligned} \phi '&=V^{-1} \circ \phi \end{aligned}$$

65 $$\begin{aligned} \phi '_{\nu '}&= \nu ' \cdot \phi ' =[( V^{-1})^t \nu '] \cdot \phi .\end{aligned}$$

66 $$\begin{aligned} \text {Therefore} \ \ A^{\phi '}_{\nu '}&= |(V^{-1})^t \nu '| A_\nu ^\phi \end{aligned}$$

67 $$\begin{aligned} \det (A^{\phi '}_{\nu '})&= |(V^{-1})^t \nu '|^n \det A^\phi _\nu \end{aligned}$$

Indeed, recall that $$V = \begin{pmatrix} V_1&\cdots&V_m \end{pmatrix}$$ is a matrix of constants and $$\phi = \begin{pmatrix} \phi _1&\cdots&\phi _m \end{pmatrix}$$ is a vector-valued function. Recall that $\phi _\nu = \nu \cdot \phi$ and the Hermitian matrix $A_\nu$ satisfies

$$\begin{aligned} \phi _\nu (z,\zeta ) = \zeta ^* A_\nu z \end{aligned}$$

where $\zeta ^*$ is the conjugate transpose of $\zeta$. Consequently, treating $\phi = \phi (z,\zeta )$ as a *vector*, $V \circ \phi ' = \phi$, and (64) follows. Consequently, since the output of $\phi$ and $\phi '$ are *vectors* in $\mathbb {C}^m$,

$$\begin{aligned} \phi '_{\nu '} = \nu '\cdot \phi ' = \nu ' \cdot (V^{-1}\phi ) = (V^{-1})^t\nu ' \cdot \phi \end{aligned}$$

and (65) holds. Next, there is no reason that $(V^{-1})^t\nu '$ is a unit vector, so we write

$$\begin{aligned} (V^{-1})^t\nu ' \cdot \phi = |(V^{-1})^t\nu '| \frac{(V^{-1})^t\nu '}{|(V^{-1})^t\nu '|} \cdot \phi = |(V^{-1})^t\nu '| \nu \cdot \phi \end{aligned}$$

using the definition of $\nu$. Consequently,

$$\begin{aligned} A_{\nu '}^{\phi '} = |(V^{-1})^t\nu '| A_{\nu }^{\phi } \end{aligned}$$

and (66) holds. Taking determinants proves (67).

Also note that $\nu ' \in \Gamma ^o_Q=\Gamma ^o_{M'}$ if and only if $\nu \in \Gamma ^o_M$. We need the following additional identities. From (63), we have

68 $$\begin{aligned} V^t \nu = \frac{\nu '}{|(V^{-1})^t \nu '|} \ \ \text {and so} \ \ \nu '_j =( V^t \nu )_j |(V^{-1})^t \nu '|, \ \ j=1, \dots , n. \end{aligned}$$

Since $\nu '$ is a unit vector, the above equality implies

69 $$\begin{aligned} |V^t \nu |= \frac{1}{ | (V^{-1})^t \nu '| } \end{aligned}$$

Now note that (63) implies

70 $$\begin{aligned} \nu ' = \frac{V^t ( \nu )}{|V^t ( \nu )|} \in S^{m-1}. \end{aligned}$$

After a calculation (see below), we obtain

71 $$\begin{aligned} d \sigma (\nu ')= \frac{|\det ( V)| \, d \sigma (\nu )}{|V^t \cdot \nu |^m}. \end{aligned}$$

where $d \sigma$ is the surface measure form on the unit sphere, $S^{m-1}$.

Now we use (61), the equalities (63)–(71) as well as $z'=z$, $V w'= w$, $\zeta '=\zeta$, and $V\eta ' = \eta$ to conclude

$$\begin{aligned} B_{\Omega '}(\alpha ', \beta ')= & {} \frac{|\det V| (2i)^m \, C_{n,m}(n+2m-1)! \ (-i)^m}{(n+m-1)!}\\{} & {} \int _{ \nu \in \Gamma ^o_{M}}\frac{\det (A^{\phi }_{\nu }) (V_1 \cdot \nu ) \dots ( V_m \cdot \nu ) \, |V^t (\nu )|^{-n-2m} \, d \sigma (\nu )}{\left[ 2 \phi '_{\nu '}(z, \zeta )+i \nu ' \cdot (w-\bar{\eta }) \right] ^{n+2m} \, |V^t (\nu )|^{-n-2m}}. \end{aligned}$$

Cancelling the common factors of $|V^t (\nu )|^{-n-2m}$ and then using (62), the proof of Theorem 6.1 is complete.

*Details on the proof of *(71). Surface measure for the unit sphere in $\mathbb {R}^m$ in the $\nu '$-rectangular coordinates is

$$\begin{aligned} d \sigma (\nu ') = \sum _{j=1}^m (-1)^{j-1} \nu '_j d \nu '_1 \wedge \cdots \wedge \widehat{j^{th}} \wedge \cdots \wedge d \nu '_m. \end{aligned}$$

The expression, $\widehat{j^{th}}$, on the right indicates that the $d \sigma '_j$ is missing.

Define the map

$$\begin{aligned} \nu ' = F(\nu ):=\frac{V^t( \nu )}{|V^t(\nu )|}, \end{aligned}$$

where $$V^t$$ is the matrix whose $$j^{th}$$ row is the vector $$V_j$$, $$1 \le j \le m$$. We will assume $$V^t $$ is orientation preserving (i.e., $$\det V = \det V^t>0$$). We wish to show

72 $$\begin{aligned} F^*(d \sigma (\nu ')) = \frac{\det (V)\, d \sigma (\nu )}{|V^t \cdot \nu |^m} \end{aligned}$$

where $V^t \cdot \nu$ indicates the product of the matrix $V^t$ with the column vector $\nu$. Denote the $j^{th}$ row of $V^t$ by $V_j=(v_{j,1}, v_{j,2}, \dots , v_{j,m}), \ j=1 \dots m$. We compute the pull back via *F*. The *d* in the expressions on the right refers to the exterior derivative in $\nu _1, \dots , \nu _m$.

73 $$\begin{aligned} F^*(d \sigma (\nu ') )&= F^* \left( \sum _{j=1}^m (-1)^{j-1} \nu '_j\,d \nu '_1 \wedge \cdots \wedge \widehat{j^{th}} \wedge \cdots \wedge d \nu '_m \right) \nonumber \\&=\sum _{j=1}^m (-1)^{j-1} \left( \frac{V_j \cdot \nu }{|V^t \cdot \nu |} \right) \left[ \frac{V_1 \cdot d \nu }{|V^t \cdot \nu |} - \frac{(V_1 \cdot \nu )d \{|V^t \cdot \nu | \}}{|V^t \cdot \nu |^2} \right] \wedge \cdots \wedge \widehat{j^{th}} \wedge \cdots \nonumber \\&\quad \wedge \left[ \frac{V_m \cdot d \nu }{|V^t \cdot \nu |} - \frac{(V_m \cdot \nu )d \{|V^t \cdot \nu | \}}{|V^t \cdot \nu |^2} \right] \nonumber \\&=\sum _{j=1}^m (-1)^{j-1} (V_j \cdot \nu ) \left( (V_1 \cdot d\nu ) \wedge \cdots \widehat{j^{th}} \wedge \cdots \wedge (V_m \cdot d\nu ) \right) |V^t \cdot \nu |^{-m} \nonumber \\&\quad - \sum _{j=1}^m (-1)^{j-1}(V_j \cdot \nu )\nonumber \\&\quad \left( \sum _{k \not =j} (V_1 \cdot d\nu ) \wedge \cdots \wedge (V_k \cdot \nu ) d \{|V^t \cdot \nu |\} \wedge \cdots \widehat{j^{th}} \cdots \wedge (V_m \cdot d \nu ) \right) \nonumber \\&\quad |V^t \cdot \nu |^{-(m+1)} \nonumber \\&= \frac{A (\nu )}{|V^t \cdot \nu |^m} - \frac{B(\nu )}{|V^t \cdot \nu |^{(m+1)}}. \end{aligned}$$

We claim $$B(\nu )=0$$. To see this, we divide up the sum over $$j \not = k $$ into two sums – one where $$j<k $$ and the other where $$j>k$$. We also move the term $$(V_k \cdot \nu ) d \{|V^t \cdot \nu |\}$$ to the very front of the wedge product, which results in an additional factor of $$(-1)^{k-1}$$ when $$k<j$$ and $$(-1)^{k-2}$$ when $$k>j$$. The difference between these two cases is due to the missing $$j^{th}$$ term when $$k>j$$. We obtain

$$\begin{aligned} B(\nu )=&\sum _{k<j} (-1)^{j-1+k-1} (V_j \cdot \nu ) (V_k \cdot \nu ) d \{|V^t \cdot \nu |\}\\&\wedge (V_1 \cdot d \nu ) \wedge \cdots \widehat{k^{th}} \wedge \cdots \widehat{j^{th}} \wedge \cdots \wedge (V_m \cdot d \nu )\\&\quad +\sum _{k>j} (-1)^{j-1+k-2}(V_j \cdot \nu ) (V_k \cdot \nu ) d \{|V^t \cdot \nu |\}\\&\wedge (V_1 \cdot d \nu ) \wedge \cdots \widehat{j^{th}} \wedge \cdots \widehat{k^{th}} \wedge \cdots \wedge (V_m \cdot d \nu ). \end{aligned}$$

By switching *j* with *k* in the second sum, and noting the sign difference, we see that these two sums cancel and so $B(\nu )=0$.

Next, we claim that $A(\nu )= \det V^t \, d \sigma (\nu )$ at each fixed $\nu _0 \in S^{m-1}$. Note that $A(\nu _0)$ belongs to the space $\Lambda ^{m-1} T^*_{\nu _0}(S^{m-1})$ which has dimension one and is spanned by $d \sigma ( \nu _0)$. So we really need to show that the coefficient of $A(\nu _0)$ in front of $d \sigma (\nu )$ is the constant, $\det V$.

Choose an orthogonal linear map $T_0: S^{m-1} \rightarrow S^{m-1}$ which maps the standard unit vector $E_1=(1, 0, \dots ,0) \in S^{m-1}$ to $\nu _0$. We apply the pull back map $T_0^*$ to $A(\nu _0)$ to obtain

$$\begin{aligned} T_0^* A(E_1)&= \sum _{j=1}^m (-1)^{j-1} (V_j \cdot T_0(E_1)) \left( (V_1 \cdot T^*_0 (d\nu )) \wedge \cdots \widehat{j^{th}} \wedge \cdots (V_m \cdot T^*_0(d\nu )) \right) . \end{aligned}$$

The result is a degree $m-1$ form at $E_1$, i.e., an element of $\Lambda ^{m-1} T^*_{E_1}(S^{m-1})$. Now note that $T_0$ is an orthogonal matrix (which preserves the inner product $\cdot$) and so

$$\begin{aligned} V_j \cdot T_0(E_1)= T_0^t ( V_j) \cdot E_1, \ \ \text {and} \ \ V_j \cdot T_0^* (d \nu ) = T_0^t (V_j) \cdot d \nu , \ \ j=1 \dots m. \end{aligned}$$

Let $V^0= T_0^t \cdot V$ (matrix multiplication). So $V^0_j = T_0^t (V_j)$ where $V^0_j$ and $V_j$ are written as column vectors for $j=1 \dots m$. Then

$$\begin{aligned} T_0^* A(E_1)=\sum _{j=1}^m (-1)^{j-1} (V^0_j \cdot E_1) \left( (V^0_1 \cdot d\nu ) \wedge \cdots \widehat{j^{th}} \wedge \cdots (V^0_m \cdot d\nu ) \right) . \end{aligned}$$

Notice that $d \sigma (\nu )$ at $E_1 \in S^{m-1}$ is just $d\nu _2 \wedge \dots \wedge d \nu _m$ (i.e., there is no $d \nu _1$). Also, let $\hat{V}_j^0 = (v^0_{j,2}, \dots v^0_{j,m}) \in \mathbb {R}^{m-1}$ and $d \hat{\nu }= (d \nu _2, \dots , d \nu _m)$. Since there is no $d \nu _1$ in $T^*_{E_1}(S^{m-1})$, we have

$$\begin{aligned} V_k^0 \cdot d\nu |_{E_1}&= \hat{V}_k^0 \cdot d \hat{\nu }, \ \ k = 1 \dots m \\&= \sum _{\ell =2}^m v^0_{k \ell } d \nu _\ell . \end{aligned}$$

Therefore

$$\begin{aligned} T_0^* A(E_1)&=\sum _{j=1}^m (-1)^{j-1} v_{j,1} \left( (\hat{V}^0_1 \cdot d \hat{\nu }) \wedge \cdots \widehat{j^{th}} \wedge \cdots (\hat{V}^0_m \cdot d\hat{\nu }) \right) . \end{aligned}$$

The wedge product on the right equals $d \hat{\nu }$ multiplied by the determinant of the $(m-1) \times (m-1)$ matrix with rows $\hat{V}_1^0, \dots , \widehat{j^{th}}, \dots \hat{V}^0_m$ (the row $\hat{V}_j^0$ is removed). This matrix is also the minor of $V^0$ with the first column and $j^{th}$ row removed. Using the formula for the determinant of $V^0$ in terms of expansion by minors, we conclude that

$$\begin{aligned} T_0^* A(E_1) = (\det V^0) \, d \hat{\nu }= (\det V^0) d \sigma (\nu )|_{E_1}. \end{aligned}$$

Since $T_0$ is orthogonal, it has determinant equal to 1, and so $\det V^0=\det V$. We conclude that $A(\nu _0)=\det V \, d \sigma (\nu _0)$, and (72) now follows.

## Data Availability

Additionally, there were no data sets used or generated by the authors for this publication.
